# Development of New CD38 Targeted Peptides for Cancer Imaging

**DOI:** 10.1007/s11307-024-01901-5

**Published:** 2024-03-13

**Authors:** Alexander Zheleznyak, Rui Tang, Kathleen Duncan, Brad Manion, Kexian Liang, Baogang Xu, Alexander Vanover, Anchal Ghai, Julie Prior, Stephen Lees, Samuel Achilefu, Kimberly Kelly, Monica Shokeen

**Affiliations:** 1 Department of Radiology, Washington University School of Medicine, St. Louis, MO 63110 USA; 2 Department of Neurosurgery, Washington University School of Medicine, St. Louis, MO 63110 USA; 3 Alvin J. Siteman Cancer Center, Washington University School of Medicine and Barnes-Jewish Hospital, St. Louis, MO 63110 USA; 4 Department of Biomedical Engineering, UT Southwestern Medical Center, Dallas, TX 75390 USA; 5 Department of Biomedical Engineering, University of Virginia, Charlottesville, VA 22908 USA; 6 Department of Biomedical Engineering, Washington University in St. Louis, St. Louis, MO 63110 USA; 7 Department of Medicine, Washington University School of Medicine, St. Louis, MO USA

**Keywords:** Multiple myeloma, CD38, Phage display, Small animal PET, Peptide bioconjugate

## Abstract

**Purpose:**

Multiple myeloma (MM) affects over 35,000 patients each year in the US. There remains a need for versatile Positron Emission Tomography (PET) tracers for the detection, accurate staging, and monitoring of treatment response of MM that have optimal specificity and translational attributes. CD38 is uniformly overexpressed in MM and thus represents an ideal target to develop CD38-targeted small molecule PET radiopharmaceuticals to address these challenges.

**Procedures:**

Using phage display peptide libraries and pioneering algorithms, we identified novel CD38 specific peptides. Imaging bioconjugates were synthesized using solid phase peptide chemistry, and systematically analyzed *in vitro* and *in vivo* in relevant MM systems.

**Results:**

The CD38-targeted bioconjugates were radiolabeled with copper-64 (^64^Cu) with100% radiochemical purity and an average specific activity of 3.3 – 6.6 MBq/nmol. The analog NODAGA-PEG4-SL022-GGS (SL022: Thr-His-Tyr-Pro-Ile-Val-Ile) had a K_d_ of 7.55 ± 0.291 nM and was chosen as the lead candidate. ^64^Cu-NODAGA-PEG4-SL022-GGS demonstrated high binding affinity to CD38 expressing human myeloma MM.1S-CBR-GFP-WT cells, which was blocked by the non-radiolabeled version of the peptide analog and anti-CD38 clinical antibodies, daratumumab and isatuximab, by 58%, 73%, and 78%, respectively. The CD38 positive MM.1S-CBR-GFP-WT cells had > 68% enhanced cellular binding when compared to MM.1S-CBR-GFP-KO cells devoid of CD38. Furthermore, our new CD38-targeted radiopharmaceutical allowed visualization of tumors located in marrow rich bones, remaining there for up to 4 h. Clearance from non-target organs occurred within 60 min. Quantitative PET data from a murine disseminated tumor model showed significantly higher accumulation in the bones of tumor-bearing animals compared to tumor-naïve animals (SUV_max_ 2.06 ± 0.4 versus 1.24 ± 0.4, P = 0.02). Independently, tumor uptake of the target compound was significantly higher (P = 0.003) compared to the scrambled peptide, ^64^Cu-NODAGA-PEG4-SL041-GGS (SL041: Thr-Tyr-His-Ile-Pro-Ile-Val). The subcutaneous MM model demonstrated significantly higher accumulation in tumors compared to muscle at 1 and 4 h after tracer administration (SUV_max_ 0.8 ± 0.2 and 0.14 ± 0.04, P = 0.04 at 1 h; SUV_max_ 0.89 ± 0.01 and 0.09 ± 0.01, P = 0.0002 at 4 h).

**Conclusions:**

The novel CD38-targeted, radiolabeled bioconjugates were specific and allowed visualization of MM, providing a starting point for the clinical translation of such tracers for the detection of MM.

**Supplementary Information:**

The online version contains supplementary material available at 10.1007/s11307-024-01901-5.

## Introduction

In multiple myeloma (MM), a cancer of malignant bone marrow plasma cells, cluster of differentiation 38 (CD38) is overexpressed. Thus, CD38 has become a valuable therapeutic target, with CD38-targeted FDA approved antibodies, daratumumab and isatuximab, being used in the clinic and new antibodies undergoing clinical trials [1]. In addition to hematological cancers, the role of CD38 is being investigated in solid cancers [2]. The differential expression (low in healthy cells, high in cancerous cells) makes CD38 a valuable target for both diagnostic imaging and therapeutic intervention.

CD38 is a 45 kDa type II transmembrane glycoprotein consisting of 300 amino acids. It is characterized by a short intracellular cytoplasmic domain, a single transmembrane helix domain, and a large extracellular domain [3]. Recent data shows that CD38 exists in two opposite membrane orientations, resulting in either extracellular or intracellular localization of the catalytic domain [4]. Additionally, through binding with the non-substrate ligand, cluster of differentiation 31 (CD31), CD38 modulates responses such as adhesion, migration, and proliferation [5]. Under physiological conditions, CD38 is expressed by premature hematopoietic cells and activated lymphocytes such as T cells, B cells, dendritic cells, and NK cells [6].

Secondary analysis of data from the Multiple Myeloma Research Foundation (MMRF) CoMMpass study showed that the CD38 expression remained high among relapsed refractory (R/R) MM patients. In 68 paired samples, we found that mean expression was higher in R/R patients (mean expression 71.2 counts per million) compared to the newly diagnosed MM (NDMM) patients (58.3 counts per million, P = 0.003) (Fig. S1). These data support the value of CD38 as a robust imaging and therapeutic target in patients with MM, even after they have relapsed or became refractory to treatment.

Encouraging progress has been made towards noninvasive molecular imaging of CD38 as antibody- and nanobody-based agents have demonstrated promising efficacy. We and others have shown that imaging of CD38 using targeted antibodies is feasible. Our group evaluated the utility of molecularly targeted PET for imaging CD38 on MM cells in preclinical mouse models using radiolabeled daratumumab, [^89^Zr]-DFO-daratumumab [7]. Caserta et al. used [^64^Cu]-daratumumab for PET imaging [8]. Ulaner et al. heralded the first-in-human [^89^Zr]-DFO-daratumumab PET in MM patients [9]. Radiolabeled daratumumab has been used to image lymphoma [10] and explored as an imaging agent in lung cancer [11], demonstrating the broad applicability of imaging CD38. Nanobodies for optical and nuclear imaging of CD38 + tumors *in vivo* have also been reported [12, 13]. In a recent study, Pape et al. reported the synthesis of a fluorochrome-conjugated nanobody that recognized a distinct non-overlapping epitope of CD38 allowing for specific detection of myeloma cells under daratumumab therapy [14].

While highly specific, antibodies are not always ideal for longitudinal imaging studies due to their relatively long biological half-life [15], and it is currently unclear if nanobodies will be widely available for clinical imaging due to challenges such as immunogenicity, production, and stability. As an alternative, high-affinity peptide-based small molecule imaging probes are comparatively less immunogenic and have favorable pharmacokinetics with robust tumor tissue uptake and rapid clearance from non-target organs *in vivo*. Additionally, peptides are straightforward to synthesize chemically, and their modularity makes them amenable to scaling for clinical use. CD38-targeted small molecule imaging agents will be ideal for longitudinal evaluation of CD38 expression during disease progression, monitoring therapeutic response, and detecting minimal residual disease. Despite their potential, there is a scarcity of CD38-targeted peptides specifically developed for imaging MM, both preclinically and clinically.

To take advantage of the above-mentioned benefits and fulfil the unmet need, we screened, identified, synthesized, and evaluated new CD38-targeted peptide bioconjugates compatible with PET imaging. Phage display is a widely recognized technology for creating and screening large libraries of peptides, with desired binding characteristics that can be used in various applications [16, 17]. We leveraged this technology to identify novel, first generation high- and low-affinity CD38-targeted peptides for developing new diagnostic probes for *in vivo* imaging [18]. Peptides were synthesized using standard solid-phase peptide synthesis techniques. Binding and specificity studies were performed with purified CD38 protein and human MM cells expressing CD38. Chelator selection for radiolabeling was based on the serum stability data and showed that the NODAGA-peptide bioconjugate was more stable than the DOTA-peptide bioconjugate for radiolabeling with ^64^Cu. The radiolabeled peptide conjugates facilitated *in vivo* and *ex vivo* tissue biodistribution and whole body noninvasive small animal PET imaging studies in intravenous (disseminated) and subcutaneous mouse models of human MM. The results suggest that the rapid clearance of the CD38-targeted small molecule imaging agents from healthy non-target tissues combined with their specific tumor uptake will enable longitudinal evaluation of CD38 expression during disease progression and monitoring of minimal residual disease after therapy, paving the way for clinical translation, and significantly improving the management of patients with MM.

## Materials and Methods

The data generated in this study are available upon request from the corresponding author.

### Reagents

^64^Cu (t_1/2_ = 12.7 h, β^+^; 17.8%, E_β+max_ = 656 keV, β^−^, 38.4%, E_β -max_ = 573 keV) was produced on a CS-15 biomedical cyclotron at Washington University School of Medicine (WUSM) with the average specific activity of 343 mCi/μg. Fluorenylmethyloxycarbonyl (Fmoc) amino acids and Rink Amide Resin were purchased from AAPPTec (Louisville, KY, USA). Dichloromethane (DCM), acetic acid, acetic anhydride, thioanisole, phenol, hydroxybenzotriazole (HOBt), HBTU (2-(1H-benzotriazol-1-yl)-1,1,3,3-tetramethyluronium hexafluorophosphate), N,N-diisopropylethylamine (DIEA), N-trityl-1,2-ethanediamine, phenol, thioanisol, dimethylformamide (DMF), N,N'-diisopropylcarbodiimide (DIC), trifluoroacetic acid (TFA), iodine, methyl tert-butyl ether (MTBE) and Chelex® 100 sodium form were purchased from Sigma-Aldrich (St. Louis, MO, USA). NODAGA-tris (t-Bu ester) was purchased from Macrocyclics, Inc (Plano, TX, USA). Milli-Q water was obtained from a Millipore Q3 system. All the acetate buffers were prepared with Chelex®-treated milli-Q water. All other chemicals were purchased from Sigma Aldrich unless otherwise noted.

### Phage display screening and selection of CD38-specific peptides

Using phage display and purified human CD38 protein (hCD38), the first generation high- and low-selectivity CD38-targeted peptides were identified for the development of CD38-targeted imaging agents [17]. Briefly, two rounds of phage display screening using the PHASTpep platform identified selective peptides for human CD38. CD38 (R&D, Catalog 2404-AC-010, Minneapolis, MN, U.S.A.) or negative control proteins, human CD4 (hCD4), human CD8 (hCD8), and GST-6His were adsorbed on Maxisorp plates (Nalgene, Nunc #442,404) according to NEB phage display protocols (NEB, Ipswich, MA). A 10 µL aliquot of PhD7 library (7 amino acid linear library) (2 × 10^11^ phage) (NEB, #E8211S) diluted in blocking buffer (DPBS/1% bovine serum albumin (BSA)) was added to each well. After 1 h of incubation at room temperature, wells were washed five times with blocking buffer and the remaining bound phage eluted into 100 μL of glycine buffer (0.2 M glycine, 0.5 M NaCl, pH 2.2) for 9 min before immediately neutralizing with 17 μL of 1 M Tris–HCl (pH 9.2).

For analysis, sequencing of the selected phage clones was performed as described in Brinton et al. [18]. Samples sent to the UVA Biomolecular Research Core Facility were deep sequenced on an Illumina MiSeq Sequencer. FASTQ files were analyzed using PHASTpep [18]. PHASTpep analyzed files were aligned for amino acid sequence homology using the Smith—Waterman algorithm.

### Generation of CD38-targeted peptides

Novel CD38 peptide sequences identified using phage display were synthesized via standard Fmoc chemistry [19]. Briefly, using the conjugated peptide NODAGA-PEG4-Thr-His-Tyr-Pro-Ile-Val-Ile-Gly-Gly-Ser-Lys-NH_2_ (NODAGA-PEG4-SL022-GGS) as an example, the following protocol was used for all peptides synthesized and subsequent bioconjugation in this study. The NODAGA-PEG4-SL022-GGS peptide was prepared using a CEM Liberty Blue microwave peptide synthesizer (Matthews, NC, USA) on Rink Amide resin. The resin (0.1 mmol) was swelled in DCM for 1 h before use. Fmoc amino acids (0.5 mmol, 5 eq), coupling reagent (HBTU, 0.5 mmol, 5 eq), and DIEA (1 mmol, 10 eq) were added to the resin and the mixture was reacted for 15 min under microwave irradiation (100W, 90 °C). The resin was washed three times with DMF. Deprotection of the Fmoc group was carried out by treatment of the resin with a solution of 20% piperidine/DMF for 5 min under microwave irradiation (100W, 90 °C). Subsequently, NODAGA-tris(t-Bu ester) (3 eq) was conjugated to the peptide while still on solid support in the presence of HOBT (5 eq), HBTU (5 eq), and DIEA (6 eq) in DMF to produce the NODAGA-PEG4-SL022-GGS peptidyl resin. The peptide was released from the resin using a cleavage cocktail of TFA: thioanisol: phenol: water (85:5:5:5, v/v/v/v) for 90 min at room temperature. The cleaved peptide product was concentrated *in vacuo* before performing reverse-phase HPLC purification (Gilson, Middleton, WI, USA). The final product’s NODAGA-PEG4-SL022-GGS (1775 Da) molecular weight was confirmed via electrospray ionization mass spectrometry with peaks observed at 889 (M + 2/2) and 593 (M + 3/3). All other peptide conjugates were synthesized, purified, and characterized in an equivalent manner.

### Radiolabeling of NODAGA-PEG4-SL022-GGS with the positron emitter ^64^Cu

^64^Cu chloride (^64^CuCl_2_) (5 − 10 µL in 0.5 M HCl) was diluted with 0.1 M ammonium acetate buffer (pH 5.5, 50 − 100 µL). NODAGA-PEG4-SL022-GGS (20 µg, 11.3 nmol) was dissolved in 200 µL ammonium acetate buffer (pH 5.5) to which buffered ^64^Cu solution (37 MBq, 1 mCi) was subsequently added. The reaction mixture was agitated during incubation at 70 °C for 15 min with slight shaking. The radiochemical purity (RCP) of the ^64^Cu-labeled NODAGA-PEG4-SL022-GGS (^64^Cu-NODAGA-PEG4-SL022-GGS) was confirmed by analytical radio-reversed phase high performance liquid chromatography (HPLC). Radiochemical reaction progress and purity were monitored using analytical radio-HPLC performed on an Agilent 1260 Infinity (Agilent Technologies, Santa Clara, CA) with a LabLogic Flow-Ram Radio HPLC detector (Lablogic Systems, Sheffield, UK). Finally, a Kinetex 5 µm XB-C18 100A 150 × 4.6 mm LC column was used, and the peptide was eluted with a gradient from 95:5 0.1% TFA in water: 0.1% TFA in CH_3_CN to 10∶90 0.1% TFA in Water:0.1% TFA in CH_3_CN over the course of 10 min. Radioactive samples were counted using a Beckman 8000 automated well-type gamma-counter (Beckman Coulter, Franklin Lakes, NJ).

### Generation of human MM.1S CD38 knockout cells

CD38 knockout (KO) cells were generated by the Genome Engineering & Stem Cell Center (GESC@MGI) at Washington University in St. Louis. Briefly, synthetic gRNA targeting the sequence 5’- CATCCTGAGATGAGGTGGGTNGG was purchased from IDT, complexed with Cas9 recombinant protein, and transfected into the MM.1S-CBR-GFP cells. Transfected cells were sorted into 96-well plates, and clones were identified using NGS to analyze out-of-frame indels for KO.

### CD38 expressing and CD38 knockout human myeloma MM.1S cell line culture

Human myeloma MM.1S cells were initially obtained from American Type Culture Collection (ATCC, Manassas, VA). MM.1S cells were modified to express Click Beetle Red (CBR) luciferase and Green Fluorescent Protein (GFP) (MM.1S-CBR-GFP) by the DiPersio laboratory (Professor John F. DiPersio, Department of Medicine, WUSM, St Louis, USA) in 2014. The expression of CD38 on the MM.1S-CBR-GFP cells (MM.1S-CBR-GFP-WT) was previously confirmed by our lab with *ex vivo* flow cytometry. Cells tested negative for mycoplasma and were analyzed by the Washington University Genome Engineering and induced Pluripotent Stem Cell Core via MycoAlert PLUS Mycoplasma Detection Kit (Lonza, Visp, Switzerland) in 2018 and 2021, and via MycoStrip Mycoplasma Detection Kit (InvivoGen, San Diego, CA) in 2022. Cell lines were passaged 4–5 times after thawing before use in *in vitro* and *in vivo* studies. Cells were routinely cultured in Roswell Park Memorial Institute (RPMI) 1640 medium (ThermoFisher Scientific, Waltham, MA) supplemented with 10% heat inactivated fetal bovine serum (FBS) (Sigma—Aldrich, St. Louis, MO) and 1% penicillin/streptomycin (Gibco, Pittsburgh, PA) at 37 °C with 5% CO2 in a humidified environment. CD38-knockout human myeloma MM.1S clones (MM.1S-CBR-GFP-KO) were developed and validated by the Genome Engineering and Stem Cell Center (GESC) at WUSM. Cell culture conditions for the MM.1S-CBR-GFP-KO cell line were consistent with those of MM.1S-CBR-GFP-WT cell line.

### Western immunoblot assays

Cells were mechanically dissociated from tissue culture flasks using rubber-tipped cell scrapers (Sarstedt, Newton, NC) and washed three times with DPBS. Cells were incubated with 25 mM Tris–HCl pH 7.6, 150 mM NaCl, 1% NP-40, 1% sodium deoxycholate, 0.1% Sodium Dodecyl Sulfate (SDS) buffer (RIPA lysis buffer, Abcam, Cambridge, MA) supplemented with EDTA-free (ethylenediaminetetraacetic acid disodium salt hydrate) protease inhibitor cocktail (Pierce, ThermoFisher, Waltham, MA) for 60 min at 4 °C followed by centrifugation at 12,000 × g for 10 min. Protein content was quantified using the bicinchoninic acid protein assay (BCA, Pierce, ThermoFisher Scientific, Waltham, MA). Lysates (10–15 µg total protein) were denatured for 5 min at 100 °C and proteins were separated by non-reducing sodium dodecyl sulfate polyacrylamide gel electrophoresis (SDS-PAGE) at 100 V for 1.5 h using Mini-PROTEAN TG precast gels with Tris/Glycine/SDS buffer system (Bio-Rad, Hercules, CA). Proteins were transferred to a polyvinylidene fluoride membrane (PVDF, Millipore, Burlington, MA) at 50 V for 2 h. After blocking for 1 h in Tris buffered saline solution supplemented with 0.05% Tween-20 and 3% BSA (TBST blocking buffer, 10 mM Tris–HCl, 150 mM NaCl, and 0.05% [v/v] Tween-20, pH 7.5, 5% (w/v) non-fat milk or 3% BSA (w/v)), the PVDF membrane was rinsed once with TBST and incubated overnight at 4 °C with 1° antibody diluted in TBST blocking buffer. After two 5-min washes, the membrane was incubated with horseradish peroxidase (HRP)-conjugated secondary antibodies in TBST blocking buffer for 1 h at room temperature. Unbound secondary antibodies was removed with three washes in TBST, 10 min each. Bound secondary antibodies were detected using SuperSignal™ West Pico Plus Detection Substrate (ThermoFischer Scientific, Waltham, MA) and ChemiDoc™ Imager (Bio-Rad, Hercules, CA). Primary antibodies used for immunoblotting were rabbit monoclonal anti-CD38 (1:1000, unconjugated, clone EPR4106, Abcam, Waltham, MA) and rabbit monoclonal anti-ß-Actin (1:1000, unconjugated, clone 13ES, Cell Signaling Technologies, Boston, MA). The secondary antibody used in this study was anti-rabbit IgG-HRP (1:10,000, conjugated to HRP, sc-2357, Santa Cruz Biotechnology, Dallas, TX).

### Microscale thermophoresis

Microscale thermophoresis (MST) assays determined the binding affinity of synthesized peptides to purified CD38 protein and was performed using the Monolith NT.115 (Nanotemper Technologies, Munich, Germany). Respective peptide stock samples were diluted in MST-1 buffer (50 mM Tris–HCl, pH 7.4; 150 mM NaCl; 10 mM MgCl_2_; 2% DMSO; 0.1% Tween-20; 4% SDS, 4 mM Dithiothreitol) at concentrations ranging from 0.0305 nM to 1000 nM or from 0.0305 µM to 1000 µM. Recombinant human CD38 protein (R&D, Catalog 2404-AC-010, Minneapolis, MN) was labeled with NT647 (Protein labeling kit red-maleimide, Nanotemper Technologies, Germany) and subsequently diluted to 5 nM in MST-2 buffer (50 mM Tris–HCl, pH 7.4, 150 mM NaCl, 10 mM MgCl_2_, 2% DMSO, and 0.1% Tween-20). Fluorescence was assessed in MST-2 buffer. Samples were loaded into NT.115 standard capillaries, and analysis was performed at 37 °C, 10% LED power, and 40% MST power. The K_d_ values were calculated from fragment concentration-dependent changes in normalized fluorescence (Fraction Bound) of different CD38 peptides based on the law of mass action using the MO Affinity Analysis v2.3 software.

### Flow cytometry

MM.1S-CBR-GFP-WT and MM.1S-CBR-GFP-KO cells were suspended in running buffer (DPBS with 2 mM EDTA and 0.5% BSA). Cell suspension was incubated with fluorochrome-conjugated monoclonal antibody PE/Cy7 CD38 (HIT2; BioLegend, San Diego, CA) in the dark at room temperature for 20 min. Cells were washed once with running buffer, and 7-amino-actinomycin D (7-AAD) was added to stain dead cells. Samples were run on an Attune Cytometer (ThermoFisher Scientific, Waltham, MA) and analyzed using FlowJo V10 (Tree Star, Ashland, OR), and dead cells excluded by gating out 7-AAD positive cells.

### Cell binding studies

To evaluate the specificity of the CD38 binding peptide, MM.1S-CBR-GFP-WT cells were suspended in binding buffer (Hank’s Balanced Salt Solution with 1 mM Ca^2+^ and 1 mM Mg^2+^ (HBSS + / + , Gibco, ThermoFisher, Waltham, MA) supplemented with 0.1% BSA) at 4 × 10^5^ cells per 250 µL, representing the reaction volume. The cells were incubated with 300 nM ^64^Cu-NODAGA-PEG4-SL022-GGS (3.3 MBq/nmol) in the presence of 75 µM unlabeled NODAGA-PEG4-SL022-GGS, 75 µM daratumumab, or 75 µM isatuximab at 4 °C for 60 min. The unbound ^64^Cu-NODAGA-PEG4-SL022-GGS was removed by three washes with HBSS + / + and cell associated activity was determined with WIZARD2® 2480 Automatic Gamma Counter (Perkin Elmer, Waltham, MA).

### Animal models

All animal studies were performed in accordance with the WUSM Institutional Animal and Use Committee guidelines. Mice were anesthetized for all injections, treatments, and imaging with 2% isoflurane/100% O_2_. Ten 5- to 6-week-old Fox Chase Severe Combined Immunodeficient (*scid*) beige (Charles Rivers Laboratories, Wilmington, MA, USA) or NOD *scid* gamma (NSG) (The Jackson Laboratory, Bar Harbor, ME, USA) mice were injected with 1 × 10^6^ MM.1S-CBR-GFP-WT cells, described above, in 100 μL DPBS subcutaneously or intravenously via lateral tail vein. Five additional 5- to 6-week-old NSG or Fox Chase *scid* beige tumor-naive mice served as non-tumor imaging and tissue biodistribution controls. Tumor burden was monitored weekly via bioluminescence imaging (BLI). Mice were randomized into cohorts for small animal PET and tissue biodistribution following administration of the radiolabeled CD38-targeted peptide. The MM.1S-CBR-GFP-WT cell line utilized here was confirmed to have the expected CD38 expression via immunocytochemistry. Tumor burden in mouse tissues was validated *in vivo* with BLI and *ex vivo* via histology and immunohistochemistry [20].

### *In vivo* bioluminescence and fluorescence imaging

*In vivo* BLI was performed by the Molecular Imaging Center at Washington University in St. Louis Medical School on the days indicated on an IVIS 50 (PerkinElmer, Waltham, MA) with Living Image 4.2, a 1–300 s exposure, binning 2–8, FOV 12.5 cm, f/stop1, open filter. Mice were injected intraperitoneally with D-luciferin (150 mg/kg in DPBS, Gold Biotechnology, St. Louis, MO) and imaged under isoflurane anesthesia (2% vaporized in O2). Total photon flux (photons/sec) was measured from fixed regions of interest (ROIs) over the entire mouse using Living Image 2.6. *In vivo* fluorescence imaging of GFP and SL022-GGS-LS288 (H-THYPIVI1-GGS-K(LS288)-NH2) (Fig. S2) tumor uptake 24 h after intravenous injection was performed on an Optix MX3 time-domain diffuse optical imaging system (Advanced Research Technologies, Montreal, Canada) (Fig. S3). Hair was removed prior to imaging with depilatory cream to improve transmission and imaged using isoflurane anesthesia (2% vaporized in O_2_). The images were corrected for background to minimize auto fluorescence and diet related non-specific fluorescence.

### Histologic assessment

After PET and biodistributions were concluded, the tibia, femurs, and spleen were harvested and fixed for 24–48 h in 10% neutral buffered formalin (NBF). Subsequent bone decalcification was performed with 14% EDTA, pH 7.4 in DPBS for 11–14 days until adequately decalcified. Specimens were processed, paraffin embedded, and sectioned at 5 µm thickness at the Musculoskeletal Histology and Morphometry Core at WUSM, Department of Orthopedic Surgery. Half of the paraffin bone and spleen sections were reserved for immunohistochemical (IHC) analysis, remaining sections were hematoxylin (Surgipath, Richmond, IL) and eosin (Richard-Allen Scientific, Kalamazoo, MI) (H&E) stained using standard procedures for routine histological analysis. Slides were viewed by a pathologist under experimentally blinded conditions.

### Immunocytochemistry

Immunocytochemistry of MM.1S-CBR-GFP-WT cells confirmed their positive CD38- and CD31- expression**.** 10,000 MM.1S-CBR-GFP-WT cells/mL were seeded in Falcon 8-chamber tissue culture treated glass slides (Corning, Tewksbury, MA) and incubated in Phenol-Free Dulbecco's Modified Eagle Medium (DMEM) (Gibco, ThermoFisher Scientific, Waltham, MA) supplemented with L-glutamine (Gibco, ThermoFisher Scientific, Waltham, MA) until confluent. Cells were fixed using ice cold 4% paraformaldehyde in DPBS, pH 7.4 for 10 min at room temperature, washed three times with DPBS, and permeabilized by incubating for 10 min with 0.1% Triton X-100 (Sigma-Aldrich, St. Louis, MO) in DPBS. Nonspecific binding was blocked by incubating cells with 1% BSA, 0.1% Tween 20 in DPBS for 30 min. After overnight incubation at 4 °C with anti-CD38 mouse polyclonal antibody (# PA5-95,840, Invitrogen, ThermoFisher Scientific, Waltham, MA, 1:100 dilution) or anti-CD31 mouse monoclonal antibody (JC/70A #MA5-13,188, Invitrogen, ThermoFisher Scientific, Waltham, MA, 1:50 dilution), cells were washed and incubated for 1 h at room temperature with Alexa Fluor 594- or 647-conjugated secondary goat anti-mouse IgG1 (cross-adsorbed, # A-21240, Invitrogen, ThermoFisher Scientific, Waltham, MA, 1:50 dilution) or F(ab')2-goat anti-rabbit IgG (H + L) (cross adsorbed, # A48285, Invitrogen, ThermoFisher Scientific, Waltham, MA, 1:100 dilution). Cells were subsequently counter stained for nuclei visualization in 0.1 µg/mL Hoechst 33,345 (ThermoFisher Scientific, Waltham, MA) for 1 min and coverslips mounted with fluorescence preserving ProLong Glass Anti-Fade Mountant (Invitrogen, ThermoFisher Scientific, Waltham, MA).

### Immunohistochemistry of CD38 expression

Immunohistochemistry (IHC) of the Formalin-Fixed, Paraffin Embedded (FFPE) tumor sections was performed by baking slides for 2 h at 60 °C, deparaffinizing with xylenes, and rehydrating through a graded ethanol to distilled water series. Heat-induced antigen retrieval was carried out using citrate-based Antigen Unmasking Solution (Vector Laboratories, Burlingame, CA). Tissue was permeabilized and nonspecific binding blocked using 0.2% Triton X-100 (Sigma-Aldrich, St. Louis, MO) and 8% normal goat serum (Sigma-Aldrich, St. Louis, MO) in DPBS. Slides were incubated overnight at 4 °C with anti-CD31 mouse monoclonal antibody (JC/70A #MA5-13,188, Invitrogen, 1:100 dilution) or anti-CD38 rabbit monoclonal antibody (EPR4106 ab108403, Abcam, Waltham, MA, 1:500 dilution) in 3% BSA in DPBS. After washing, Alexa Fluor 647- or 594-conjugated secondary antibodies goat anti-mouse IgG1 (cross adsorbed, # A-21240, Invitrogen, ThermoFisher Scientific, Waltham, MA, 1:100 dilution) or F(ab')2 goat anti-rabbit IgG (H + L) (cross adsorbed, # A48285, Invitrogen, ThermoFisher Scientific, Waltham, MA, 1:100 dilution) were added to the slides and incubated for 2 h in the dark. The slides were counter-stained with Hoechst 33,342 (ThermoFisher Scientific, Waltham, MA), and sealed as in ICC protocol.

### Microscopic analysis of ICC and IHC

Microscopy of ICC and IHC slides was performed on either Olympus BX51 epifluorescence microscope or an Olympus Fluoview 1000 confocal microscope (Olympus, Center Valley, PA). The Olympus BX51 epifluorescence microscope was equipped with 40x, 60x, 100 × objectives and DAPI, GFP, Texas Red, Cy5, Cy7, and 775/845 filter sets; the confocal microscope was equipped with 505, 560, 635, 705, and 785 nm lasers with 20x, 40x, 60 × objectives. Tumor images were acquired with Olympus cellSens or Cell Standard 1.6 software and exported for analysis with ImageJ software (NIH, Bethesda, MD).

### Small animal PET

PET studies were conducted at the Preclinical Imaging Facility at the Mallinckrodt Institute of Radiology, WUSM. Animals from the Fox Chase *scid* beige (5–6 weeks old males, Charles River Laboratory, Wilmington, MA) or the NSG (5–6 weeks old males, NOD *scid* gamma, Jackson Laboratory, Bar Harbor, ME) mouse strains bearing either systemically administered MM1.S-CBR-GFP-WT xenograft, a subcutaneous MM1.S-CBR-GFP-WT xenograft, or a tumor-naive control were anaesthetized under continuous Isoflurane vapor (Pivetal®, DCM, WUSM, 2% vaporized in O_2_) and injected with 6.6 MBq/nmol (1 µg) ^64^Cu-NODAGA-PEG4-SL022-GGS via lateral tail vein. Imaging was performed 1 h, 2 h, or 4 h after the injection of the indicated bioconjugates using a Mediso nanoScan PET122S/CT1520 in vivo imager (Mediso, Arlington, VA) or Siemens Inveon MM PET/CT scanner (Siemens Corporation, Washington, D.C.). Mediso coincident photons were filtered with an energy window between 400 and 600 keV, and reconstruction was performed with four full iterations, six subsets per iteration with an isotropic voxel size of 0.4 mm^3^ using the TeraTomo 3D reconstruction algorithm. CT-based attenuation correction was applied for PET reconstruction. Siemens Inveon coincidences were filtered with energy window between 350–650 keV; the reconstruction algorithm was OSEM3D/MAP with 0.8 mm^3^ voxel size and CT-based attenuation correction.

### Tissue biodistribution of ^64^Cu-NODAGA-PEG4-SL022-GGS

Biodistribution studies were conducted according to previously published protocols [21, 22]. Briefly, 6.6 MBq/nmol ^64^Cu-NODAGA-PEG4-SL022-GGS was injected via the tail vein. Animals were sacrificed at selected time points after, or after the conclusion of PET, organs of interest were harvested, weighed, and the associated radioactivity determined on a γ-counter. After correcting for background and decay, the percent-injected dose per gram (%ID/g) was calculated by comparison to a weighed and counted standard.

### Secondary analysis of Multiple Myeloma Research Foundation (MMRF) CoMMpass study data

We performed a secondary analysis of data from the MMRF CoMMpass study (Courtesy, Mark Fiala, PhD and Ravi Vij, MD). CoMMpass is a longitudinal study of over 1000 MM patients which incorporates both clinical and genomic data. As part of the CoMMpass study, RNA-sequencing (RNA-seq) on CD138-enriched bone marrow cells was performed using Illumina TruSeq RNA library kits by Translational Genomics Research Institute (TGEN). For this analysis, data were extracted from the MMRF Researcher Gateway Portal corresponding with Interim Analysis 22.

### Statistical analysis

Data was presented as mean ± SD and statistical analysis performed using GraphPad Prism Version 9.1.0 software (GraphPad Software, Inc., La Jolla, CA) and Microsoft Excel. A two-tailed unpaired t-test was used for data with two groups and one variable. Data with one variable and multiple groups were analyzed with a one-way ANOVA and Tukey's or Dunnett's multiple comparisons test to determine the adjusted P-value. Data with two variables and multiple groups were analyzed with a two-way ANOVA and Tukey's multiple comparisons test to determine the adjusted P-value. P values of less than 0.05 were considered statistically significant.

## Results

### Phage display identification of CD38 selective peptides

To identify peptides selective for CD38, we biopanned a random, 7 amino acid peptide phage library on recombinant hCD38 as well as hCD4, hCD8, and GST-His. After two rounds of biopanning on each protein, phage were harvested, amplified, and processed for next generation sequencing (NGS) [18]. FASTQ files were uploaded into PHASTpep, translated into peptides, and tables of frequency produced. All sequences were normalized to the naïve library (pre-selection) sequence frequencies and finally the fold change for each peptide frequency between hCD38 and the average of the same peptide frequency from the hCD4, hCD8, and GST-His screens was calculated. The Smith-Waterman homology algorithm was employed to identify clusters of similar peptides (Table S1). From the analysis, two clusters emerged with more than 5 peptides in the homology family and also had high selectivity (PHASTpep Score, Table S1). In both clusters, the top peptide sequence had a greater than 700- and 670-fold selectivity for hCD38 over the other proteins biopanned. Therefore, we synthesized the highest scoring peptides from both clusters, A (THYPIVI, SL022) and B (FSRDWTS, SL028). Peptide sequences with less than a tenfold selectivity score are generally non-selective and thus, we used the sequence, VNYHFPV, as a negative control.

### Molecular design and synthesis

To validate the PhastPep results, we synthesized THYPIVI (SL022) from cluster A and FSRDWTS (SL028) from cluster B with the addition of the tri-amino acid sequence, GGS, on the C terminus using standard FMOC chemistry. The flexible GGS linker provides structural flexibility and has been applied to connect functional peptide domains to favor interdomain interactions or movements [23]. The binding affinities of GGS-SL022, AC-SL022-GGS, GGS-SL028 and AC-SL028-GGS for CD38 were compared (Table S2). The binding affinity data prompted the attachment of GGS on the amino versus the C-terminus. To enable *in vivo* near infrared (NIR) fluorescence imaging, a small NIR cyanine dye (LS288) was conjugated to SL022-GGS-LS288 and SL028-GGS-LS288 ((Table S3).The radionuclide chelator DOTA modified peptide sequences were prepared for the cluster A high specificity sequence SL022 and low specificity sequence VNYHFPV (SL025) (Table S3, DOTA-SL022-GGS, DOTA-β-Ala-SL022-GGS and DOTA-β-Ala-SL025-GGS). To improve the *in vivo* stability of ^64^Cu chelation, the chelator NODAGA was used in place of DOTA for the subsequent studies (Table S3, NODAGA-SL022-GGS and NODAGA-SL025-GGS). To enhance blood circulation without compromising the binding affinity, tetra-ethylene glycol (PEG4) was incorporated into the high specificity peptide sequence to generate NODAGA-PEG4-SL022-GGS (Table S3). In addition, we prepared a PEGylated NODAGA modified cluster A peptide without GGS tag to compare the performance with NODAGA-PEG4-SL022-GGS both *in vitro* and *in vivo* (Table S3, NODAGA-PEG4-SL022).

### Microscale Thermophoresis (MST) assay to determine binding affinities

**Fig. 1:**
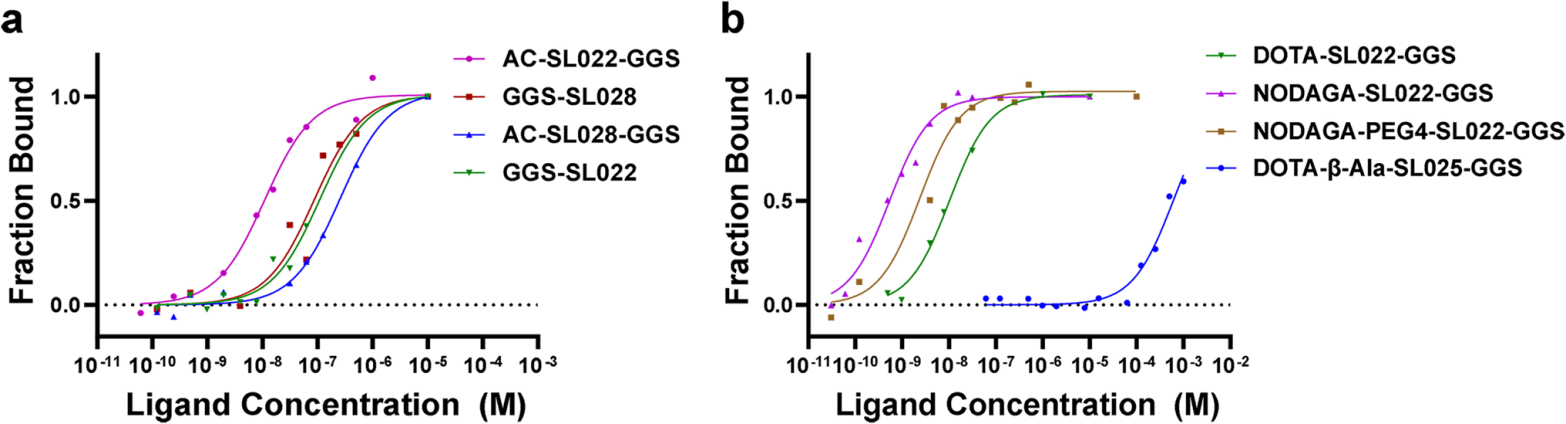
Binding studies evaluating CD38-peptides using purified CD38 protein. Microscale thermophoresis (MST) analysis of binding affinities between purified CD38 protein and various CD38 peptide sequences. Using the MST assay, which measures the tendency of macromolecules to migrate along a thermal gradient, the K_d_ was determined using Alexa-647 labeled CD38 protein; (**a**) Evaluation of binding kinetics of high scoring peptides from both cluster A and cluster B respectively; (**b**) Evaluation of chelator modified CD38 peptides.

Microscale Thermophoresis (MST) measures molecular interactions based on temperature gradient-dependent mobility of fluorescent biomolecules. We assessed the specificity and affinity of 8 CD38-targeted peptides for purified CD38 protein using the MST method. MST analysis showed nanomolar range K_d_ for all the high specificity peptides from clusters A and B (Fig. 1, Table S2). MST analysis of the interaction between CD38 peptides modified with chelators or PEG4 validated the results from the PhastPep analysis. DOTA modified SL022 has a K_d_ of 89.2 ± 1.04 nM, NODAGA modified SL022 has a K_d_ of 0.524 ± 0.127 nM and NODAGA-PEG modified SL022 has a K_d_ of 7.55 ± 0.291 nM demonstrating high specificity and affinity for binding CD38 recombinant protein (Fig. 1 and Table 1). In contrast, the predicted low specificity/affinity binding peptide SL025 had negligible binding. The K_d_ value was 605 ± 0.697 µM for DOTA-β-Ala-SL025-GGS (modified with DOTA and β-Alanine), and undetermined for NODAGA-SL025-GGS (modified with NODAGA). Based on the affinity data, serum stability results, and blood circulation kinetics, NODAGA-PEG4-SL022-GGS was the best choice for subsequent *in vitro* and *in vivo* validation studies.

**Table 1:** Binding of peptides modified with chelators for radiolabeling

Compound	Peptide	K_d_ (Mean)
DOTA-β-Ala-SL025-GGS	DOTA-β-Ala-Val-Asn-Tyr-Hdis-Phe-Pro-Val-Gly-Gly-Ser-Lys-NH_2_ (VNYHFPV)	605 ± 0.697 µM
DOTA-SL022-GGS	DOTA-Thr-His-Tyr-Pro-Ile-Val-Ile-Gly-Gly-Ser-Lys-NH_2_ (THYPIVI)	89.2 ± 1.04 nM
NODAGA-SL022-GGS	NODAGA-Thr-His-Tyr-Pro-Ile-Val-Ile-Gly-Gly-Ser-Lys-NH_2_ (THYPIVI)	0.524 ± 0.127 nM
NODAGA-PEG4-SL022-GGS	NODAGA-PEG4-Thr-His-Tyr-Pro-Ile-Val-Ile-Gly-Gly-Ser-Lys-NH_2_ (THYPIVI)	7.55 ± 0.291 nM
NODAGA-SL025-GGS	NODAGA-Lys-Val-Asn-Tyr-His-Phe-Pro-Val-Gly-Gly-Ser-NH_2_ (VNYHFPV)	ND

### Radiolabeling the CD38 bio-conjugates

**Fig. 2:**
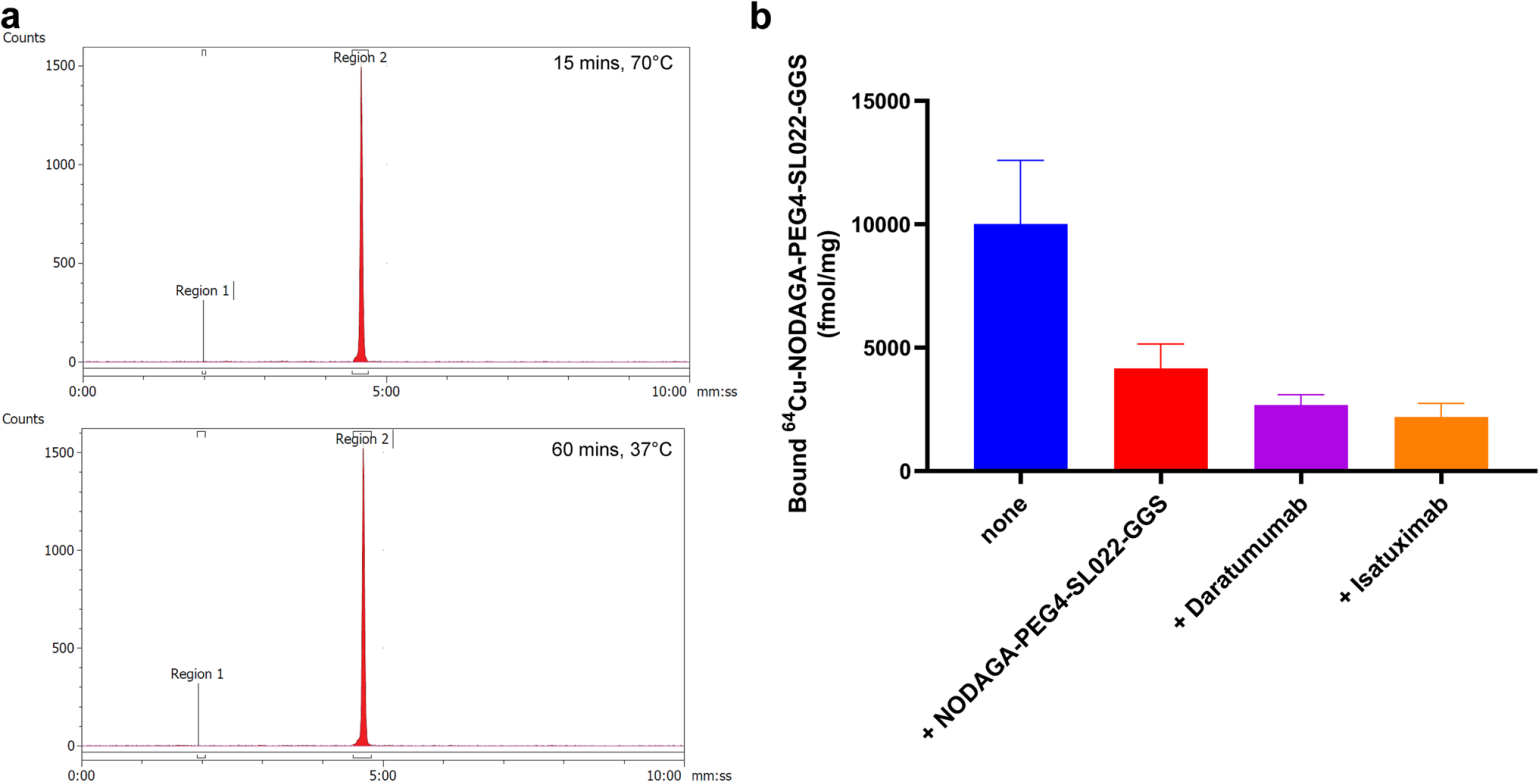
Radiolabeling and cellular uptake studies (**a**) Representative chromatograph of the ^64^Cu labeled CD38-peptide (NODAGA-PEG4-SL022-GGS) under two different conditions; (**b**) *In vitro* blocking study of ^64^Cu-NODAGA-PEG4-SL022-GGS in MM.1S-GFP-CBR-WT cells with unlabeled NODAGA-PEG4-SL022-GGS, daratumumab and isatuximab respectively.

The CD38-targeted peptide bioconjugate, NODAGA-PEG4-SL022-GGS, was successfully radiolabeled with ^64^Cu resulting in a specific activity (SA) of 3.3 MBq/nmol – 6.6 MBq/nmol (N = 10). The radiochemical purity (99 ± 0.03%) was confirmed by analytical radio-HPLC (Fig. 2a). Serum stability studies demonstrated the robustness of ^64^Cu-NODAGA-PEG4-SL022-GGS for up to 4 h (Fig. S4). The NODAGA-PEG4-SL022-GGS labeling capacity was investigated using labeling reactions with increasing amounts of ^64^Cu. NODAGA-PEG4-SL022-GGS could be radiolabeled *up to* a maximum SA of ~ 148 MBq/nmol with 100% radiochemical purity (Fig. S5). Overall, the radiolabeling studies demonstrated robust labeling capacity of NODAGA-PEG4-SL022-GGS.

#### NODAGA-PEG4-SL022-GGS retains its affinity and specificity for binding CD38 when expressed in its native cell membrane context

 In order to determine whether the conjugates were able to identify CD38 protein expressed in its native context, we performed cell binding assays with MM.1S-CBR-GFP-WT cells. For DOTA-β-Ala-SL025-GGS the K_d_ was calculated as 16.6 and 15.9 nM (N = 2, triplicates). However, this molecule was not pursued due to the serum instability of the copper labeled molecule (^64^Cu-DOTA-β-Ala-SL025-GGS). The ability of NODAGA-PEG4-SL022-GGS to bind specifically to CD38 expressed on the surface of MM.1S-CBR-GFP-WT human MM cells was assessed in an *in vitro* system. NODAGA-PEG4-SL022-GGS was radiolabeled with ^64^Cu with 3.3 MBq/nmol SA. MM.1S-CBR-GFP-WT cells were allowed to bind 300 nM ^64^Cu-NODAGA-PEG4-SL022-GGS for 60 min at 4 °C in the presence or absence of 75 µM of either unlabeled NODAGA-PEG4-SL022-GGS, 75 µM daratumumab, or 75 µM isatuximab. As seen in Fig. 2b, 250-fold molar excess of unlabeled NODAGA-PEG4-SL022-GGS lead to about 2-fold decrease in cell-bound ^64^Cu-NODAGA-PEG4-SL022-GGS (0.4 × 10^4^ ± 0.2 × 10^4^ fmol/mg versus 1 × 10^4^ ± 0.6 × 10^4^ fmol/mg, P = 0.029). Unlabeled daratumumab and isatuximab were also effective at preventing the binding of ^64^Cu-NODAGA-PEG4-SL022-GGS to CD38 expressed by MM.1S-CBR-GFP-WT cells (0.3 × 10^4^ ± 0.06 × 10^4^, P = 0.037 for daratumumab, 0.2 × 10^4^ ± 0.09 × 10^4^, P = 0.04 for isatuximab). Furthermore, there was about 68% reduced uptake of ^64^Cu-NODAGA-PEG4-SL022-GGS in the MM.1S-CBR-GFP-KO cells as compared to the MM.1S-CBR-GFP-WT cells (P < 0.006) (Fig. S6a). Overall, the cell binding data showed that ^64^Cu-NODAGA-PEG4-SL022-GGS bound to CD38 in a specific manner.

### Histological characterization of MM.1S-CBR-GFP-WT *in vitro* and *in vivo*

**Fig. 3:**
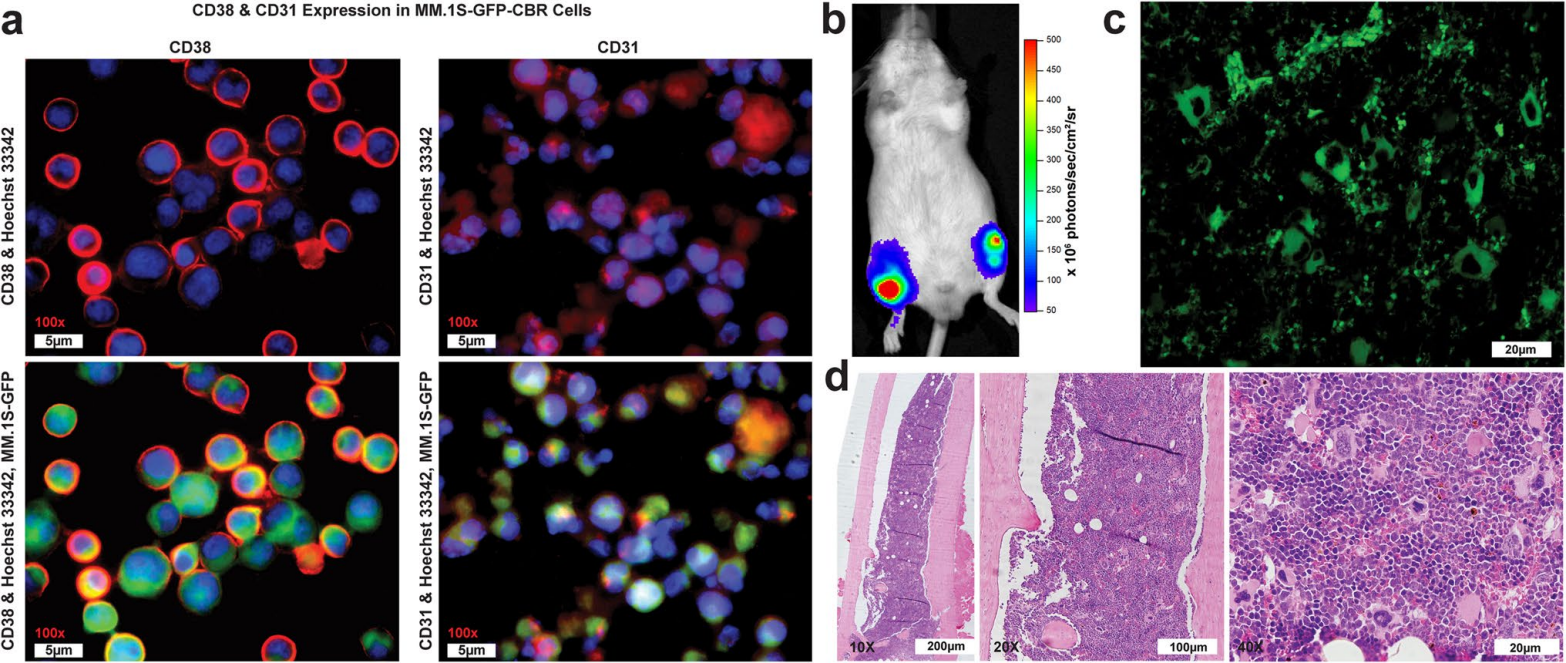
**a** CD38 & CD31 expression in MM.1S-GFP-CBR-WT cells. CD38 (left column): Immunocytochemistry analysis of CD38 expression in MM.1S-CBR-GFP cells with the CD38 1° antibody followed by the AlexaFluor 647 conjugated goat anti-rabbit IgG secondary antibody. Nuclei were counter stained with Hoechst 33,342. The sections were imaged with GFP filter pair (410/520) for endogenous GFP expression, Cy5 (620/705) for CD38 with AlexaFlour 647-conjugated secondary antibody, and Hoechst 33,342 (370/405) to stain nuclei. CD31 (right column): Immunohistochemistry analysis of CD31 expression in MM.1S-CBR-GFP cells with CD31 antibody followed with AlexaFluor 647 conjugated goat anti-rabbit IgG secondary antibody. Nuclei were counter-stained with Hoechst 33,342. The sections were imaged with GFP filter pair (410/520) to visualize endogenous GFP expression, Cy5 (620/705) for CD31 expression with AlexaFlour 647-conjugated secondary antibody, Hoechst 33,342 (370/405) to stain nuclei; **b** Characterization of the disseminated MM.1S-CBR-GFP-WT tumor model. *In vivo* BLI showing presence of disseminated MM.1S-CBR-GFP-WT in both femurs and tibia at 5 weeks after tumor implantation; **c** MM.1S-CBR-GFP-WT tumor burden additionally evidenced in the femur via GFP intracellular expression seen with epifluorescence microscopy of unstained femur section, scale bar – 20 µm; **d** Histological assessment of the femur with H&E staining shown at 10x, 20x, 40 × at scale of 200 µm, 100 µm, 20 µm, respectively.

To confirm the MM.1S-CBR-GFP-WT human MM cell line as a CD38 positive MM mouse model for CD38-targeted therapies, internal GFP reporter expression within MM.1S-CBR-GFP-WT human MM cell line was demonstrated *via* immunocytochemistry and immunohistochemistry. MM.1S-CBR-GFP-WT cells were incubated with anti-CD38 and anti-CD31 antibodies and imaged showing high expression of both CD38 and CD31 on the cell membrane and intracellularly. The membrane associated CD38 exhibited high expression on over 80% of MM.1S-CBR-GFP-WT cells (Fig. 3a). CD31 was also shown to be highly expressed. Additionally, the expression of CD38 by MM.1S-CBR-GFP-WT was examined by flow cytometry (Fig. S6b) and Western blotting (Fig. S7). These data demonstrated that CD38 expression by MM.1S-CBR-GFP-WT was higher than that of A549 cell line (human lung carcinoma) [24] and U87-MG cell line (human glioblastoma) [25], both known expressors of CD38. MM.1S-CBR-GFP-KO cell line showed no expression of CD38 by either western blot or flow cytometry. The disseminated disease model was effective at establishing the disease state, as confirmed with BLI (Fig. 3b). The tumor-bearing mice were sacrificed, femur bones extracted, and immunohistochemistry performed to demonstrate *in vivo* presence of CD38 + MM tumor burden. Unstained femur section showed GFP intracellular expression under epifluorescence microscopy indicating MM.1S-CBR-GFP-WT tumor burden in the femur (Fig. 3c). Additionally, H&E staining showed the presence of the myeloid cells while CD38 antibody staining indicated the presence of CD38 + cells (Fig. 3d).

### Tissue biodistribution and PET

**Fig. 4:**
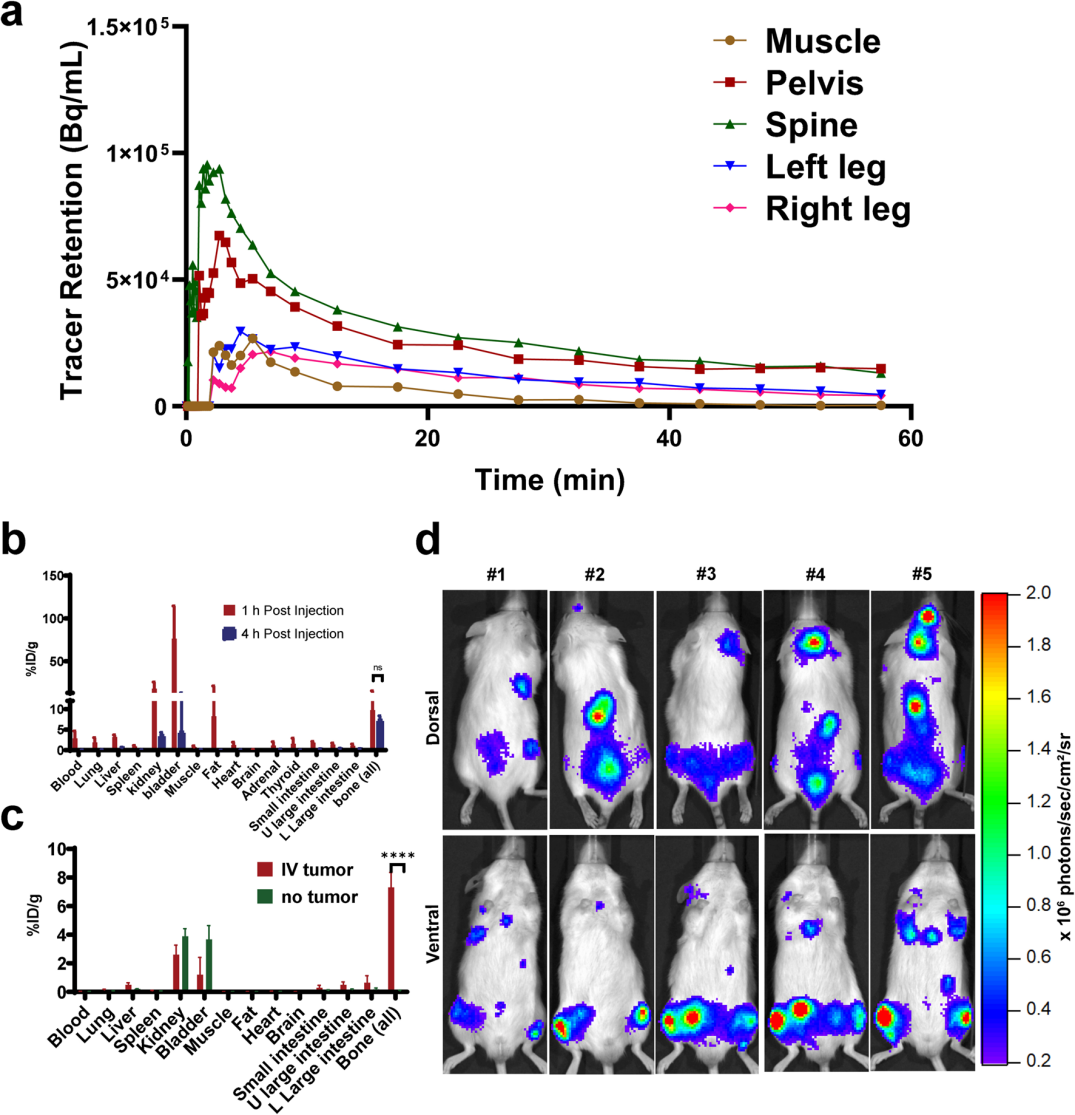
**a** Analysis of real-time ^64^Cu-NODAGA-PEG4-SL022-GGS accumulation (time-activity curve, TAC) in muscle, leg bones, spine, and pelvis of Fox Chase *scid* beige male mice (N = 3) bearing systemically implanted MM1.S-CBR-GFP-WT xenografts. The mice were injected with 6.6 MBq ^64^Cu-NODAGA-PEG4-SL022-GGS coincidental with the start of the PET scan. Disintegrations were collected from 0 to 60 min. Disintegration coincidences were filtered with energy window between 350–650 keV. Reconstruction algorithm was OSEM3D/MAP, with 0.8 mm^3^ voxel size. CT based attenuation correction was used. VOI were identified in lower extremities, spine, pelvis, and muscles. Kinetic analysis was performed using the Inveon Research Workstation software and the tracer accumulation was plotted against the time of acquisition. Statistical analysis (GraphPad Prizm) details: spine versus muscle P < 0.0001; pelvis versus muscle P < 0.0001; left leg versus muscle (10 – 60 min) P = 0.003; right leg versus muscle (10 – 60 min) P = 0.006; **b** Tissue biodistribution in tumor-bearing mice at 1 and 4 h after tracer administration. Fox Chase *scid* beige male mice (5 – 6 weeks old) were systemically implanted with 1 × 10^6^ MM1.S-CBR-GFP-WT human MM cells via lateral tail vein (N = 10). Tumor growth progression was monitored with BLI weekly. When the total flux reached 10^6^ photons/sec, animals were injected with 6.6 MBq/nmol ^64^Cu-NODAGA-PEG4-SL022-GGS via the lateral tail vein. Animals were sacrificed at 1 h (N = 5) and 4 h (N = 4) after tracer administration, organs of interest were harvested, weighed, and the associated radioactivity determined using a γ-counter. After correcting for background and decay, the percent-injected dose per gram (%ID/g) was calculated by comparison to a weighed, counted standard; **c** Tissue biodistribution at 4 h after administering ^64^Cu-NODAGA-PEG4-SL022-GGS. Fox Chase *scid* beige male mice (5 – 6 weeks old) were systemically implanted with 1 × 10^6^ MM1.S-CBR-GFP-WT human MM cells via lateral tail vein (N = 5). Control mice (N = 5) did not receive implants. Tumor growth progression was monitored with BLI weekly. When the total flux reached 10^6^ photons/sec, tumor-bearing and control mice were injected with 6.6 MBq/nmol.^64^Cu-NODAGA-PEG4-SL022-GGS via the lateral tail vein. Animals were sacrificed 4 h after tracer administration, organs of interest were harvested, weighed, and the associated radioactivity determined using a γ-counter. After correcting for background and decay, the %ID/g was calculated by comparison to a weighed, counted standard. P = 0.00003; **d** BLI showing that MM.1S-CBR-GFP-WT human MM cells injected intravenously localize to the bone marrow rich skeletal sites (spine and femurs).

The distribution of a tracer immediately after administration is critical to understanding its *in vivo* behavior. Accordingly, we investigated the dynamic *in vivo* distribution of the tracer during the first 60 min after the administration. We established the disseminated disease in Fox Chase *scid* beige mice (N = 3) by systemically implanting MM.1S-CBR-GFP-WT human MM cells *via* lateral tail vein injection and performed the dynamic PET of ^64^Cu-NODAGA-PEG4-SL022-GGS. The tissue uptake and retention were analyzed in leg, pelvis, and spine then compared to tracer retention in the muscle, a non-target tissue. The time-activity curves (TAC) representing the retention in these tissues are shown in Fig. 4D and Table S4. These data demonstrated that a significant amount of activity was detected in spine (5.05 × 10^4^ Bq/mL) and pelvic bones (2.27 × 10^4^ Bq/mL) immediately after the administration compared to the activity in muscle (0.53 × 10^4^ Bq/mL), P < 0.0001 and P < 0.0009, respectively. This significant difference in retention was maintained throughout the data acquisition. Specifically, spine retained 4.35 × 10^4^ Bq/mL, pelvis retained 2.18 × 10^4^, while muscle retained 0.47 × 10^4^ Bq/mL. Although the ^64^Cu-NODAGA-PEG4-SL022-GGS uptake in the leg bones was not different from that in muscle during 0 to 10 min, the tracer amount was significantly higher in leg bone versus muscle from 10 min until the end of data acquisition (left leg versus muscle: 1.14 × 10^4^ versus 0.39 × 10^4^ Bq/mL, P = 0.003; right leg versus muscle: 1.0 × 10^4^ versus 0.39 × 10^4^ Bq/mL, P = 0.006). These data demonstrate that ^64^Cu-NODAGA-PEG4-SL022-GGS targeted and retained in marrow-rich bones for the duration of the study.

As a small peptide, ^64^Cu-NODAGA-PEG4-SL022-GGS was expected to be cleared from the body relatively quickly. Therefore, we compared the *ex-vivo* biodistribution of ^64^Cu-NODAGA-PEG4-SL022-GGS at 1 h and 4 h after tracer administration. The data in Fig. 4b demonstrated rapid clearance of ^64^Cu-NODAGA-PEG4-SL022-GGS from non-target organs through the kidneys 1 h after tracer administration while being retained in the target organs. Complete clearance of ^64^Cu-NODAGA-PEG4-SL022-GGS from non-target organs was achieved at 4 h after tracer administration, with 80% decrease in the kidney (18 ± 7.7%ID/g versus 3.6 ± 0.7%ID/g) and 94% decrease in the bladder (76 ± 38%ID/g versus 4.4 ± 8.7%ID/g). In contrast, the tracer accumulation in bone was retained with only 25% decrease at the same 4 h time point (9.8 ± 5.7%ID/g versus 7.3 ± 1.04%ID/g). Importantly, the difference in bone retention between 1 and 4 h after tracer administration was not significant. We compared the tumor-bearing and tumor-naïve animals to show that ^64^Cu-NODAGA-PEG4-SL022-GGS was retained in the marrow rich skeletal sites of tumor-bearing compared to tumor-naïve animals (Fig. 4c and Table S5). As expected, the bones of tumor-bearing animals retained significantly more ^64^Cu-NODAGA-PEG4-SL022-GGS than the tumor-naïve controls (6.9 ± 1.03%ID/g versus 0.1 ± 0.06%ID/g, P = 0.00003). The *in vivo* and *ex vivo* biodistribution data together demonstrated that ^64^Cu-NODAGA-PEG4-SL022-GGS accumulated in the marrow rich bones within minutes of injection and cleared through the kidneys slower than it did from the non-target organs. The retention of ^64^Cu-NODAGA-PEG4-SL022-GGS in target organs was significantly higher than the retention in non-target organs at 1 and 4 h after tracer administration and did not accumulate in the marrow rich bones of tumor naïve animals.

**Fig. 5:**
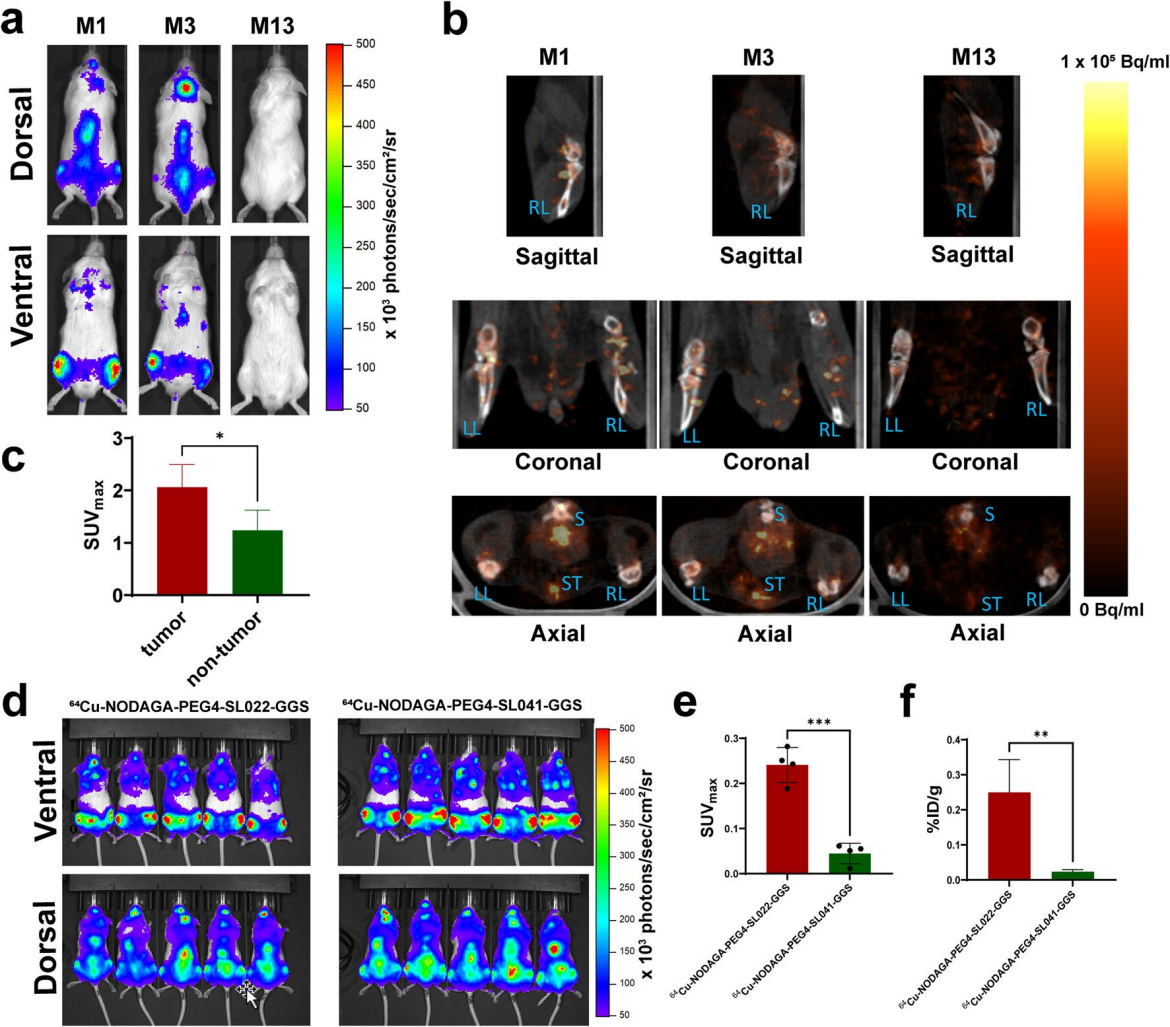
**a** BLI demonstrating the tumor burden prior to PET; **b** Representative PET of MM disseminated xenografts with ^64^Cu-NODAGA-PEG4-SL022-GGS. NSG (NOD *scid* gamma) mice (N = 10) were systemically implanted with 1 × 10^6^ MM1.S-CBR-GFP human MM cells via lateral tail vein. Control mice (N = 5) did not receive implants. The tumor growth progression was monitored with BLI. ^64^Cu-NODAGA-PEG4-SL022-GGS (6.6 MBq/nmol per animal in saline) was injected via tail vein 14 days after tumor implantation. Disintegration coincidences were collected for 20 min 1 h after tracer administration and static images were reconstructed with four full iterations, six subsets per iteration with an isotropic voxel size of 0.4 mm^3^ using the TeraTomo 3D reconstruction algorithm. Top images: coronal and sagittal projections of M1 and M3 tumor bearing and M13 non-tumor bearing control animals in prone positions. Bottom images: axial projections. S – spine, ST – sternum, RL – right leg, LL – left leg. The signal was equally scaled for all images (0 – 1 × 10^5^ Bq/ml). BLI images acquired 13 days after tumor implantation. The signal was equally scaled for all images (50 – 500 × 10^3^ photons/sec/cm^2^/sr); **c** SUV_max_ obtained from quantification of PET. P = 0.02; **d** NSG (NOD *scid* gamma) mice (N = 10) were systemically implanted with 1 × 10^6^ MM1.S/CBR/GFP human MM cells via lateral tail vein. Bioluminescence images (BLI) obtained 5 weeks after tumor implantation demonstrating the tumor burden prior to PET. Animals were injected IV with either ^64^Cu-NODAGA-PEG4-SL022-GGS (N = 5) or ^64^Cu-NODAGA-PEG4-SL041-GGS (N = 5) at 6.6 MBq /nmol per animal in saline. Disintegration coincidences were collected for 20 min 2 h after tracer administration and static images were reconstructed with four full iterations, six subsets per iteration with an isotropic voxel size of 0.4 mm^3^ using the TeraTomo 3D reconstruction algorithm; **e** SUV_max_ obtained from quantification of PET images; **f** Animals were euthanized at the conclusion of PET acquisition, leg bones were excised, and associated activity was quantified as %ID/g. P values corrected for false discovery rate (FDR).

Next, we investigated the behavior of ^64^Cu-NODAGA-PEG4-SL022-GGS in a disseminated tumor model to better understand the tracer’s performance against background accumulation in tissues expressing the endogenous target receptor. The disseminated MM model was generated in immunocompromised animals through intravenous implantation of MM.1S-CBR-GFP-WT human MM cells (N = 10). The disease progression was monitored by imaging the bioluminescent signal from the CBR reporter in MM.1S-CBR-GFP-WT cells (Fig. 5a). After establishing the diseased state, the animals were imaged with ^64^Cu-NODAGA-PEG4-SL022-GGS/PET 1 h after tracer administration. Representative PET images in Fig. 5b showed that ^64^Cu-NODAGA-PEG4-SL022-GGS accumulated in marrow rich bones (femur, tibia, spine, and pelvis) of tumor-bearing animals. The data in Fig. 5c showed the maximum standard uptake values (SUV_max_) quantified in the bones demonstrated the tracer accumulation was significantly higher when compared to the tumor naïve animals (N = 5) (SUV 2.06 ± 0.4 versus 1.24 ± 0.4, P = 0.02). ^64^Cu-NODAGA-PEG4-SL022-GGS accumulation in the bones of tumor-naïve animals was expected and was likely due to the normal expression of CD38 by the cells of hematopoietic lineage present in the bone marrow. To further illustrate the *in vivo* specificity of ^64^Cu-NODAGA-PEG4-SL022-GGS, we compared its target organ accumulation to that of the non-specific “scrambled” peptide, ^64^Cu-NODAGA-PEG4-SL041-GGS (SL041: Thr-Tyr-His-Ile-Pro-Ile-Val) (Table S3). We implanted MM.1S-CBR-GFP-WT MM cells in NSG mice (N = 10). After the tumors were established (Fig. 5d), animals were injected with either ^64^Cu-NODAGA-PEG4-SL022-GGS (N = 5) or ^64^Cu-NODAGA-PEG4-SL041-GGS (N = 5) at 6.6 MBq/nM per animal. PET was performed 2 h after the tracers’ administration. The data in Fig. 5e and Fig. S8 demonstrated significant ^64^Cu-NODAGA-PEG4-SL022-GGS accumulation in leg bones of the animals compared to that of the non-specific ^64^Cu-NODAGA-PEG4-SL041-GGS peptide (SUV_max_ 0.24 ± 0.04 versus SUV_max_ 0.05 ± 0.02, P = 0.0001). When the leg bones were excised after the completion of PET and accessed for the associated activity, the ^64^Cu-NODAGA-PEG4-SL022-GGS showed significantly higher accumulation compared to the non-specific peptide (Fig. 5f, 0.25 ± 0.09%ID/g versus 0.02 ± 0.006%ID/g, P = 0.003). Overall, PET data demonstrated the *in vivo* validity of ^64^Cu-NODAGA-PEG4-SL022-GGS as a CD38-specific PET imaging agent.

We further investigated the performance of ^64^Cu-NODAGA-PEG4-SL022-GGS as an imaging agent in subcutaneous model of MM. Subcutaneous MM.1S-CBR-GFP-WT xenografts were generated in immunocompromised animals (N = 3). After the tumors reached 200 mm^3^ (approximately), the animals were imaged with ^64^Cu-NODAGA-PEG4-SL022-GGS at 1 and 4 h after administration. The representative images in Fig. S9a and S9b showed that ^64^Cu-NODAGA-PEG4-SL022-GGS accumulated and was retained in tumors for 4 h. SUV_max_ were significantly higher than background (muscle) at both time points, (0.8 ± 0.3 for bone and 0.1 ± 0.04 muscle, P = 0.04 at 1 h and 0.9 ± 0.01 for bone and 0.1 ± 0.01 for muscle, P = 0.0002 at 4 h) (Fig. S9c). As anticipated, clearance through kidneys decreased at 4 h after tracer administration. The difference in ^64^Cu-NODAGA-PEG4-SL022-GGS retention in tumors at both time points was not significant, which suggested that the compound remained in tumors despite the kidney clearance.

## Discussion

This work was motivated by the benefits that peptides provide, such as modularity, scalability, favorable pharmacokinetics, and low immunogenicity [26]. Small peptide conjugates can be designed to give effective nuclear or optical readouts of cell surface or intracellular proteins; these probes can also potentiate imaging of dynamic cellular processes [27]. Specifically, we focused on identifying, designing, synthesizing, and evaluating peptide conjugates specific for CD38, which is a clinically viable target for MM and other pathologies. Phage display is a versatile technology designed to screen peptides that bind to a specific target with high affinity [28]. Utilizing phage display and pioneering algorithms, we identified novel peptides that were selective for CD38 [18]. These selective (high scoring) peptide sequences were synthesized using the standard solid phase peptide chemistry. We synthesized 13 analogs for downstream *in vitro* and *in vivo* evaluations. Binding characteristics were evaluated using diverse assays. Overall, the binding data for the homology clusters was in alignment with the selectivity predictions identified by the phage display approach.

The human myeloma MM.1S-CBR-GFP-WT cell line was utilized here for its expression of CD38 in 96.1% of MM.1S cells [20]. Our *in vivo* studies demonstrated the presence of CD38 positive cells in murine bone marrow as well as in immunofluorescence imaging of bone sections performed *ex vivo*. To the best of our knowledge, this is the first report of characterizing CD38 expression in the disseminated MM.1S model.

The significant functional associations within the transmembrane adhesion receptors of CD38 make a targeted radiolabeled peptide well-suited for imaging and therapy. While the MST assay provided accurate representation of ligand-receptor interaction, it did not consider the critical *cis* interactions between CD38 and the neighboring molecules in the plane of the cell membrane. We used the cell binding and blocking studies to investigate whether the *cis* environment affected the affinity of NODAGA-PEG4-SL022-GGS bioconjugate to the membrane expressed CD38. Interestingly, both daratumumab and isatuximab (clinically approved anti-CD38 antibodies) demonstrated efficient blocking capabilities along with the non-radioactive peptide conjugates. Although isatuximab and daratumumab do not share the binding site on CD38, Lee et al. compared the crystal structures of both antibodies in complex with CD38 noting that there was steric collision between the VH chain of isatuximab and the VL chain of daratumumab [29]. It can be argued that the binding epitope for SL022 is near the antibodies’ epitopes creating a strong possibility for the steric hindrance phenomenon. In our future studies, we will evaluate the spatial interaction of these new CD38 peptides with the CD38 epitopes.

Finally, *in vivo* data demonstrated the targeting specificity of the CD38 peptides for the CD38 expressed on the human MM cells in the disseminated and subcutaneous xenograft models. Specificity of retention was demonstrated in diverse *in vivo* conditions, including the comparison with the non-specific scrambled ^64^Cu-NODAGA-PEG4-SL041-GGS peptide, where the target ^64^Cu-NODAGA-PEG4-SL022-GGS peptide was shown to illuminate the MM-containing bones. The ^64^Cu-NODAGA-PEG4-SL022-GGS peptide accumulated in disease-bearing bones versus the bones of tumor-naïve animals. Significantly higher tracer uptake was demonstrated in tumor-bearing skeletal tissues such as spine, pelvis, and legs even though CD38 was known to be expressed at low levels in various tissues [6, 30]. As expected, the rapid blood clearance and excretion *via* kidneys was apparent. Our next phase of studies will involve enhancing tumor uptake and retention *via* multimerization of the high affinity peptides, while retaining rapid clearance through kidneys. Furthermore, these optimizations will be evaluated in therapeutic settings to image the effects on CD38 expression following CD38-targeted therapy as well as other potent myeloma therapies. In addition to the advantages of using novel peptide-based systems, our goal is to develop peptides that do not interfere with the epitopes that are engaged by the therapeutic antibodies such as daratumumab [31, 32].

### Supplementary information

Below is the link to the electronic supplementary material.

Supplementary file1 (PDF 692 KB)

## Data Availability

The authors declare that the data supporting the findings of this study are available within the paper and its Supplementary Information files. Should any raw data files be needed in another format they are available from the corresponding author upon reasonable request.
