# Supplementary material for: Development of New CD38 Targeted Peptides for Cancer Imaging
Source: Mol Imaging Biol. Author manuscript; Available in PMC 2024 Aug 1. (PMC11282151; doi:10.1007/s11307-024-01901-5)
Supplement: Supplementary Material [file NIHMS1986742-supplement-Supplementary_Material.pdf]

Electronic Supplementary Material

Development of New CD38 Targeted Peptides for Cancer Imaging

Alexander Zheleznyak, Rui Tang, Katie Duncan, Brad Manion, Kexian Liang, Baogang Xu, Alexander Vanover, Anchal Ghai, Julie Prior, Stephen Lees, Samuel Achilefu, Kimberly Kelly and Monica Shokeen*

List of Content:

1. **Table S1**. CD38 targeted sequences identified by phage display.
2. **Table S2**. Microscale thermophoresis (MST) analysis of binding affinities between purified CD38 protein and various CD38 peptide sequence.
3. **Table S3**. Bio-conjugate design and characteristics.
4. **Fig. S1**. Normalized mRNA expression from CD138-selected bone marrow cells from paired-samples of MM patients (N = 68).
5. **Fig. S2**. Absorption and fluorescence spectra of NIR-dye conjugated CD38 targeted peptides SL022-GGS-LS288 in PBS.
6. **Fig. S3**. In vivo NIR fluorescence imaging of SL022-GGS-LS288 in a subcutaneous MM.1S-CBR-GFP tumor in a SCID mouse model.
7. **Fig. S4**. Serum stability study of ^{64}Cu-NODAGA-PEG4-SL022-GGS.
8. **Fig. S5**. ^{64}Cu radiolabeling of NODAGA-PEG4-SL022-GGS.
9. **Fig. S6**. Binding of ^{64}Cu-NODAGA-PEG4-SL022-GGS to MM.1S cells either expressing (WT) or not expressing (KO) CD38 and CD38 expression on the surface of MM.1S-CBR-GFP-WT and MM.1S-CBR-GFP-KO analyzed by flow cytometry.
10. **Fig. S7**. Western blot representing expression of CD38 by MM.1S-CD38-WT, MM.1S-CD38-KO, A549, and U87-MG cells.
11. **Table S4.** Biodistribution of the probe at 1 hour and 4 hour time points.
12. **Table S5.** Biodistribution of the probe in tumor bearing and tumor naïve mice at 4 hour time point.
13. **Fig. S8.** Representative PET comparing target compound with the scrambled control in a disseminated MM.1S-CBR-GFP-WT xenograft model.
14. **Fig. S9.** Representative PET obtained in a subcutaneous MM.1S-CBR-GFP-WT human MM xenograft mouse model.

Cluster A		Cluster B	
Sequence	Score	Sequence	Score
THYPIVI	730.1	FSRDWTS	671.1
TPYPIVL	429.7	YDWTMHS	299.7
VSYHFPV	194.4	YKDWSEW	266.4
DLVHYPE	1.5	MPVARDW	2.1
VNYHFPV	1.2	QGTDWTM	1.7
		QTSHDWL	1.7

Table S1. CD38 targeted sequences identified by phage display. Clusters A and B were identified from phage display heat maps and anonymized data based on the Smith-Waterman local alignment clustering algorithm. The clusters/sequences were significantly enriched for CD38 compared to 6xHis (control), hCD4, and hCD8.

Compound Label	K_d-Mean (nmol/L)	K_d-SD (nmol/L)	Response Amplitude
GGS-SL022	104.1	174.8	7.88
AC-SL022-GGS	8.57	2.25	4.91
GGS-SL028	82.59	52.94	1.97
AC-SL028-GGS	243.46	88.88	4.64

Table S2. Microscale thermophoresis (MST) analysis of binding affinities between purified CD38 protein and various CD38 peptide sequence: K_d values (**Figure 1a**).

Compound Label	M/W	Sequence	Active sequence	ClogP*	Design
**GGS-SL022**	1170	H-Lys-Gly-Gly-Ser-Thr-His-Tyr-Pro-Ile-Val-Ile-NH_2	THYPIVI	-2.19	high score cluster A
**AC-SL022-GGS**	1213	AC-Thr-His-Tyr-Pro-Ile-Val-Ile-Gly-Gly-Ser Lys-NH_2	THYPIVI	-2.23	high score cluster A
**SL022-GGS-LS288**	2125	H-Thr-His-Tyr-Pro-Ile-Val-Ile-Gly-Gly-Ser-Lys(LS288)-NH_2	THYPIVI	-17.17	high score cluster A, fluorescent tagged
**GGS-SL028**	1226	H-Lys-Gly-Gly-Ser-Phe-Ser-Arg-Asp-Trp-Thr-Ser-NH_2	FSRDWTS	-8.93	high score cluster B
**AC-SL028-GGS**	1269	AC-Phe-Ser-Arg-Asp-Trp-Thr-Ser-Gly-Gly-Ser-Lys-NH_2	FSRDWTS	-8.75	high score cluster B
**SL028-GGS-LS288**	2181	H-Phe-Ser-Arg-Asp-Trp-Thr-Ser-Gly-Gly-Ser-Lys(LS288)-NH2	FSRDWTS	-15.52	high score cluster B, fluorescent tagged
**DOTA-SL022-GGS**	1556	DOTA-Thr-His-Tyr-Pro-Ile-Val-Ile-Gly-Gly-Ser Lys-NH_2	THYPIVI	-6.83	high score cluster A, DOTA modified
**DOTA-β-Ala-SL022-GGS**	1627	DOTA-β-Ala-Thr-His-Tyr-Pro-Ile-Val-Ile-Gly-Gly-Ser-Lys-NH2	THYPIVI	-7.20	high score cluster A, DOTA-β-Ala modified
**NODAGA-SL022-GGS**	1527	NODAGA-Thr-His-Tyr-Pro-Ile-Val-Ile-Gly-Gly-Ser Lys-NH_2	THYPIVI	-5.62	high score cluster A, NODAGA modified
**NODAGA-PEG4-SL022-GGS**	1775	NODAGA-PEG4-Thr-His-Tyr-Pro-Ile-Val-Ile-Gly-Gly-Ser-Lys-NH_2	THYPIVI	-6.44	high score cluster A, NODAGA-PEG4 modified
**DOTA-β-Ala-SL025-GGS**	1661	DOTA-β-Ala-Val-Asn-Tyr-His-Phe-Pro-Val-Gly-Gly-Ser-Lys-NH_2	VNYHFPV	-8.33	low score cluster A, DOTA-β-Ala modified
**NODAGA-SL025-GGS**	1561	NODAGA-Lys-Val-Asn-Tyr-His-Phe-Pro-Val-Gly-Gly-Ser-NH_2	VNYHFPV	-6.85	low score cluster A, NODAGA modified
**NODAGA-PEG4-SL022**	1446	NODAGA-PEG4-Ile-Val-Ile-Pro-Tyr-His-Thr-NH_2	THYPIVI	-3.73	high score cluster A, NODAGA-PEG4 modified
**NODAGA-PEG4-SL041-GGS**	1775	NODAGA-PEG4-Thr-Tyr-His-Ile-Pro-Ile-Val-Gly-Gly-Ser-Lys-NH_2	TYHIPIV	-6.44	Scrambled peptide for high score cluster A, NODAGA-PEG4 modified

*** CLogP value is calculated in the ChemDraw software v. 20.0.0.41(PerkinElmer)**

Table S3. Bioconjugate design and characteristics. Peptide conjugates were synthesized to identify the sequences exhibiting optimal imaging agent features based on the phage display screening and scores.

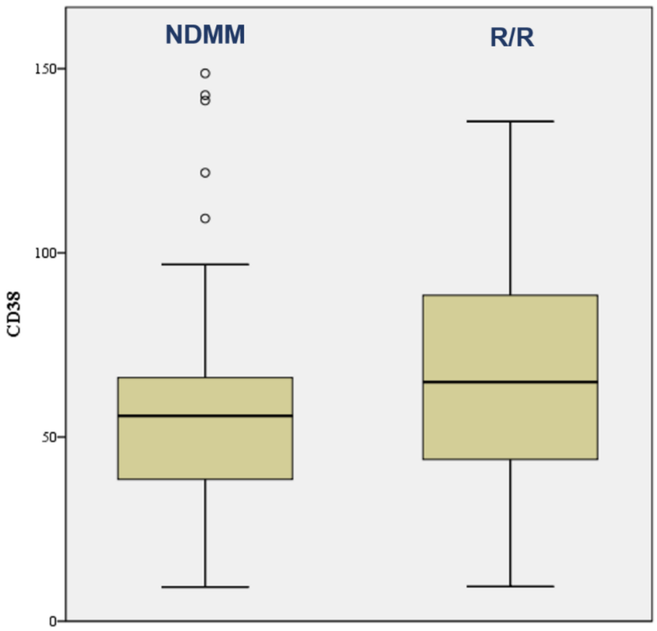

Fig. S1. Normalized mRNA expression from CD138-selected bone marrow cells from paired-samples of MM patients (N = 68). CD38 expression remains high among relapsed refractory patients. NDMM: Newly Diagnosed Multiple Myeloma. R/R: Relapsed/Refractory

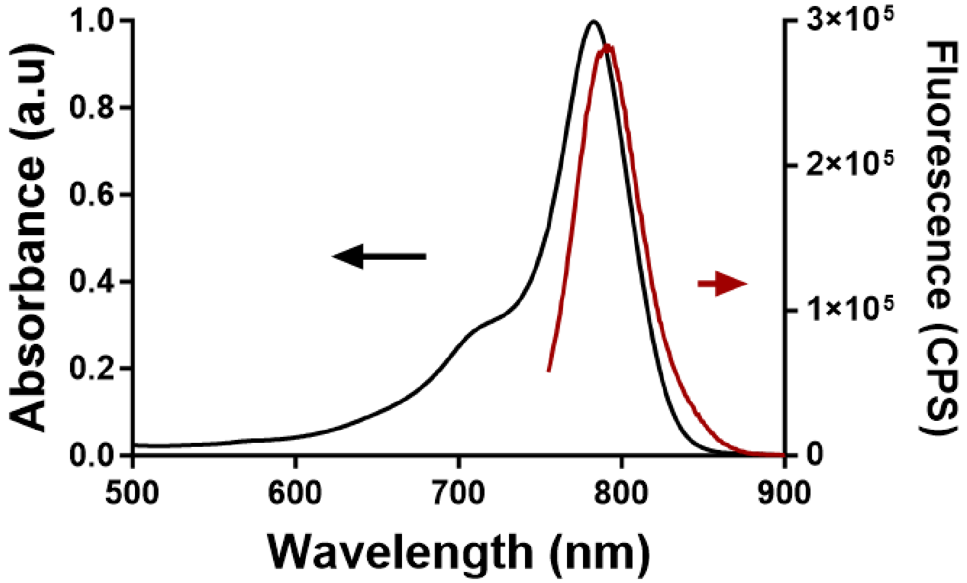

Fig. S2. Absorption and fluorescence spectra of SL022-GGS-LS288 in PBS.

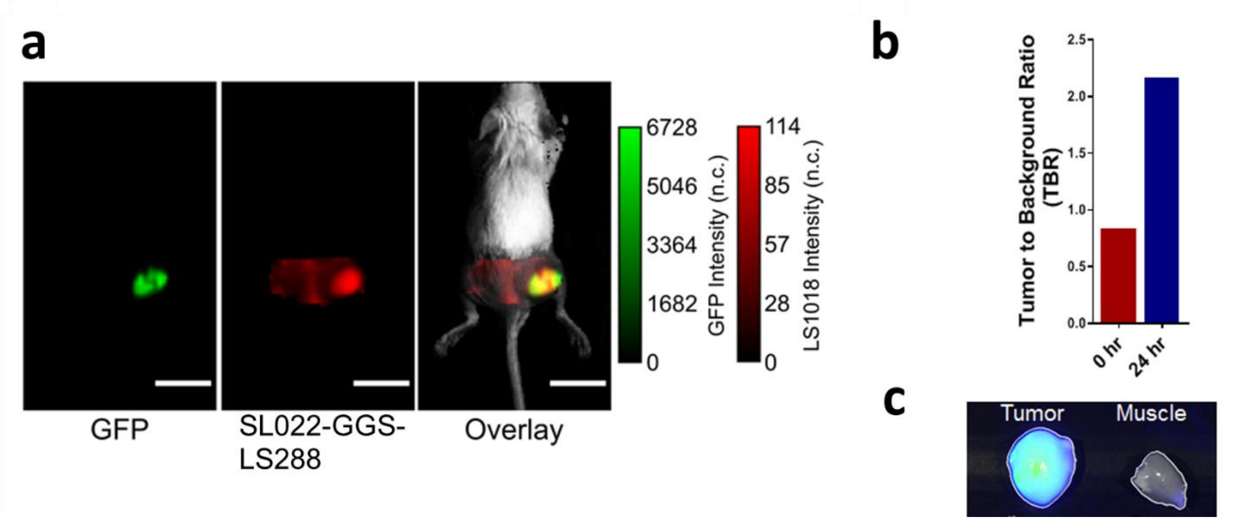

Fig. S3. a) Green fluorescent protein (GFP) and SL022-GGS-LS288 (H-THYPIVI-GGS-K(LS288)-NH_2) fluorescent images of tumor uptake at 24 hours after tracer administration. Images were taken on the Optix MX3 time-domain diffuse optical imaging system (Advanced Research Technologies; n.c., normalized counts). **b)** SL022-GGS-LS288 achieved a tumor-to-background ratio of approximately 2.5 24 hours after tracer administration (tumor and contralateral ROI analysis of the image shown in a). **c)** *Ex vivo* biodistribution data showed an 8.79 tumor to muscle ratio.

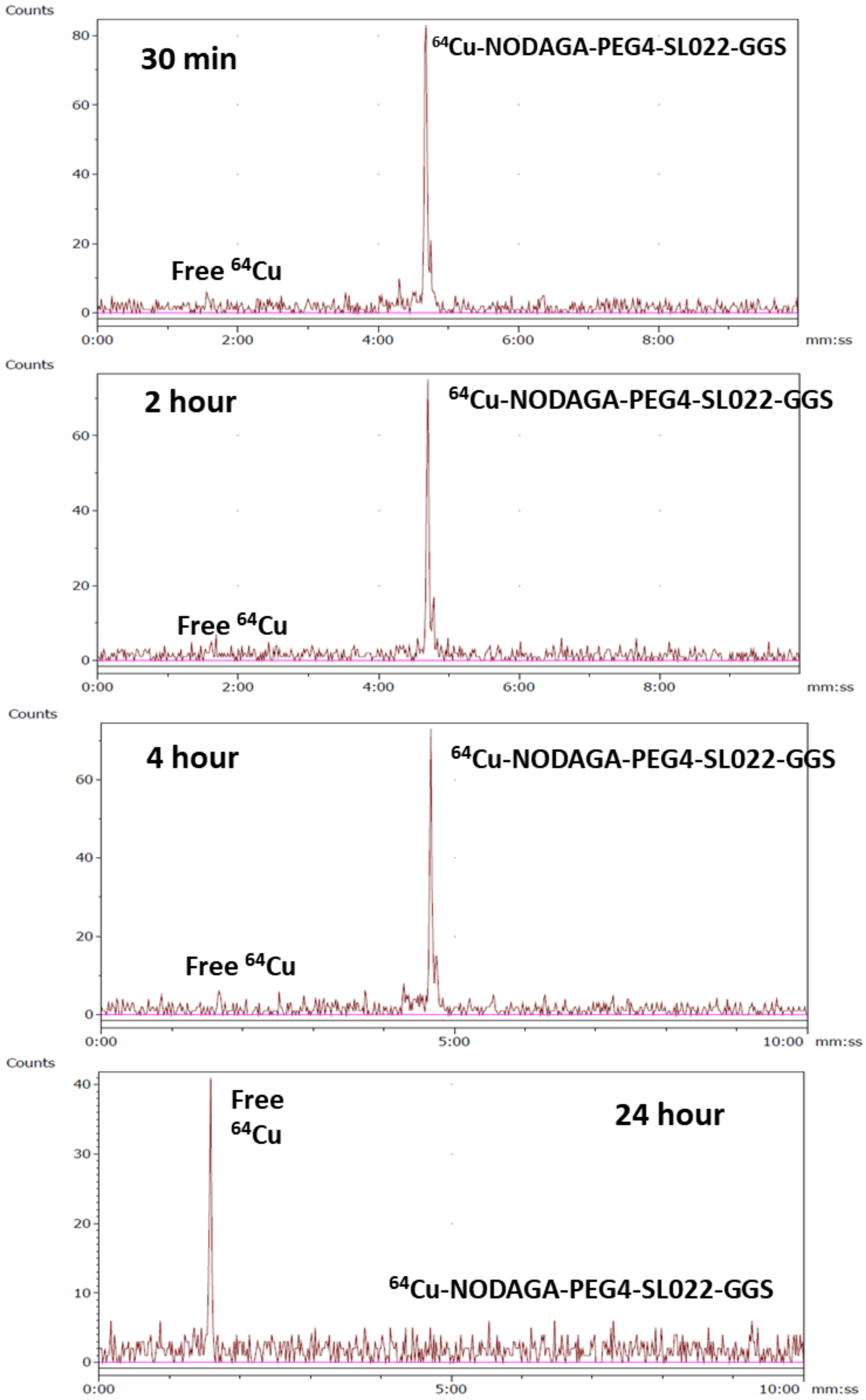

Fig. S4. Analytical radio-HPLC assessment of ^{64}Cu-NODAGA-PEG4-SL022-GGS incubated in mouse serum at 37°C for 24 hours. Aliquots were collected at 0.5, 2, 4, and 24 hours.

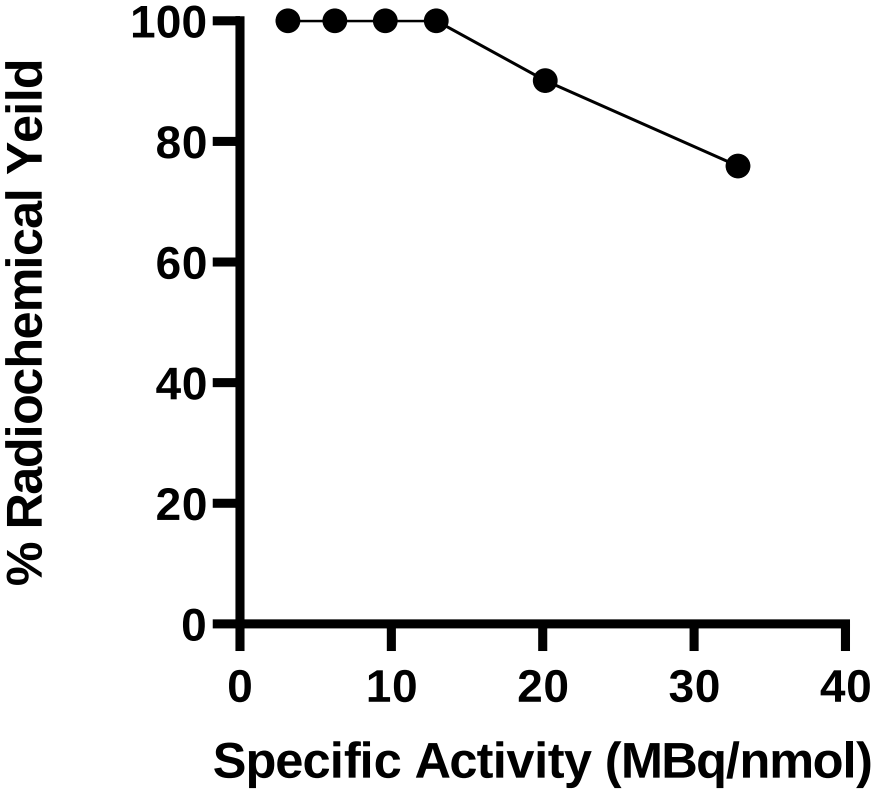

Fig. S5. ^{64}Cu radiolabeling of NODAGA-PEG4-SL022-GGS. 20 µg of NODAGA-PEG4-SL022-GGS was incubated with a range of ^{64}Cu radioactivity (37 – 370 MBq) for 15 minutes at 70°C.

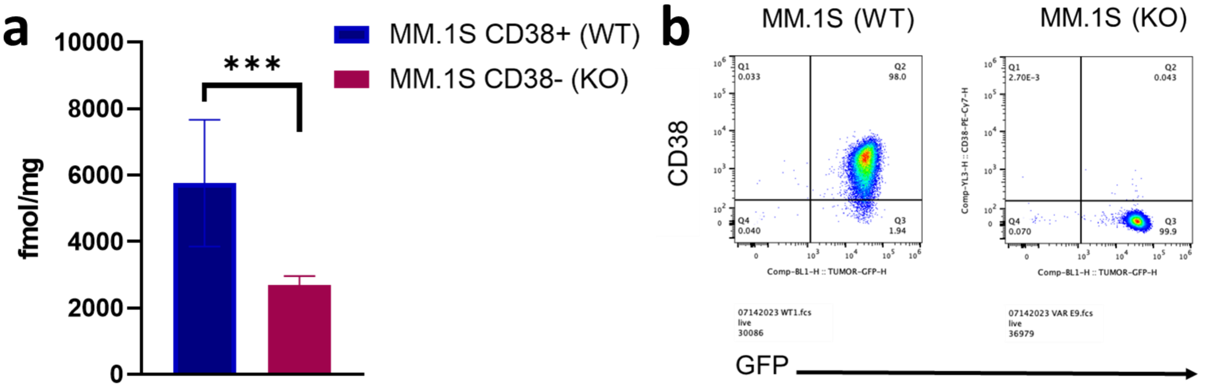

Fig. S6. a) Binding of ^{64}Cu-NODAGA-PEG4-SL022-GGS to MM.1S-CBR-GFP-WT cells either expressing (WT) or not expressing (KO) CD38, $P < 0.006$. **b)** CD38 expression on the surface of MM.1S-CBR-GFP-WT and MM.1S-CBR-GFP-KO analyzed by flow cytometry.

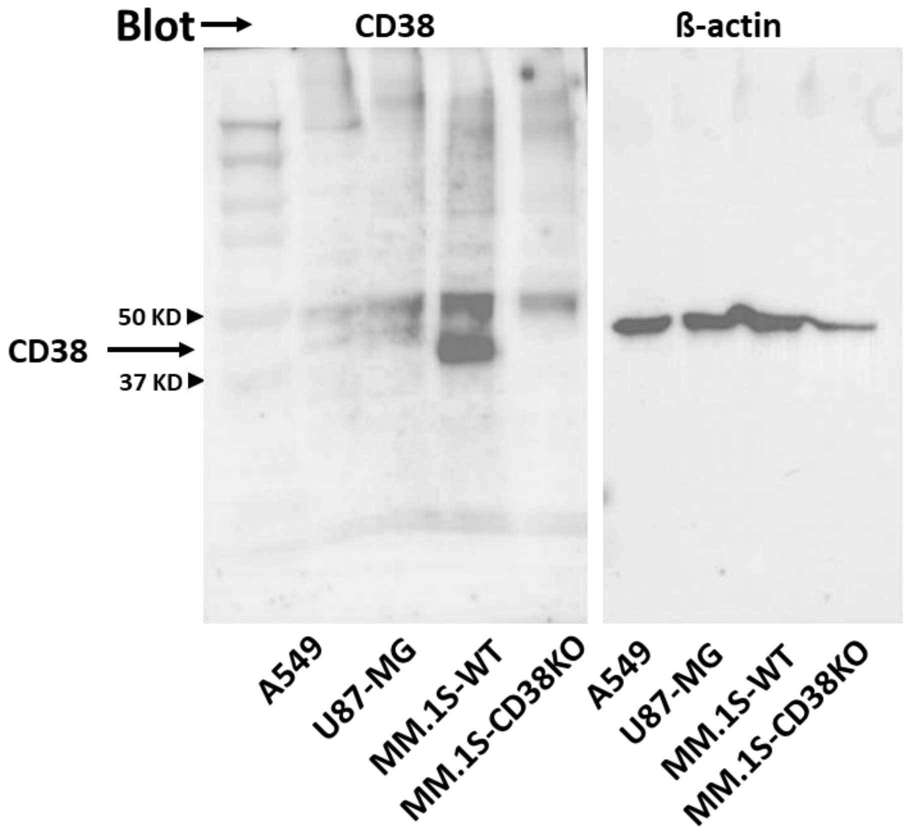

Fig. S7. Western blot representing expression of CD38 by MM.1S-CBR-GFP-WT, MM.1S-CBR-GFP-KO, A549, and U87-MG cells.

	1 hour (N = 4)		4 hour (N = 4)	
	%ID/g	SD	%ID/g	SD
Blood	2.87	1.80	0.04	0.03
Lung	1.90	1.15	0.12	0.03
Liver	3.21	0.49	0.60	0.14
Spleen	0.93	0.21	0.10	0.03
kidney	17.99	7.73	3.57	0.74
bladder	76.46	38.03	4.42	8.67
Muscle	0.77	0.26	0.07	0.12
Fat	8.27	12.84	0.02	0.02
Heart	1.23	0.69	0.06	0.03
Brain	0.12	0.04	0.01	0.00
Adrenal	1.14	0.91	0.11	0.17
Thyroid	1.57	1.34	0.03	0.02
Small intestine	1.84	0.28	0.26	0.07
U large intestine	1.38	0.33	0.34	0.15
L Large intestine	1.08	0.41	0.31	0.06
Bone	9.77	5.66	7.31	1.04

Table S4: Biodistribution of ^{64}Cu-NODAGA-PEG4-SL022-GGS at 1 hour and 4 hour time points.

	Tumor bearing (N = 4)		Tumor naïve (N = 4)	
	%ID/g	SD	%ID/g	SD
Blood	0.03	0.01	0.02	0.01
Lung	0.15	0.04	0.07	0.01
Liver	0.50	0.14	0.20	0.02
Spleen	0.08	0.03	0.06	0.01
kidney	2.60	0.67	3.89	0.53
bladder	1.21	1.20	3.67	0.97
Muscle	0.01	0.00	0.02	0.01
Fat	0.01	0.01	0.01	0.00
Heart	0.04	0.01	0.02	0.00
Brain	0.01	0.00	0.01	0.00
Small intestine	0.29	0.17	0.11	0.04
U large intestine	0.50	0.20	0.16	0.04
L Large intestine	0.64	0.49	0.18	0.09
Bone	6.91	1.03	0.07	0.05

Table S5: Biodistribution of ^{64}Cu-NODAGA-PEG4-SL022-GGS in tumor-bearing mice and tumor-naïve mice at 4 hour timepoint.

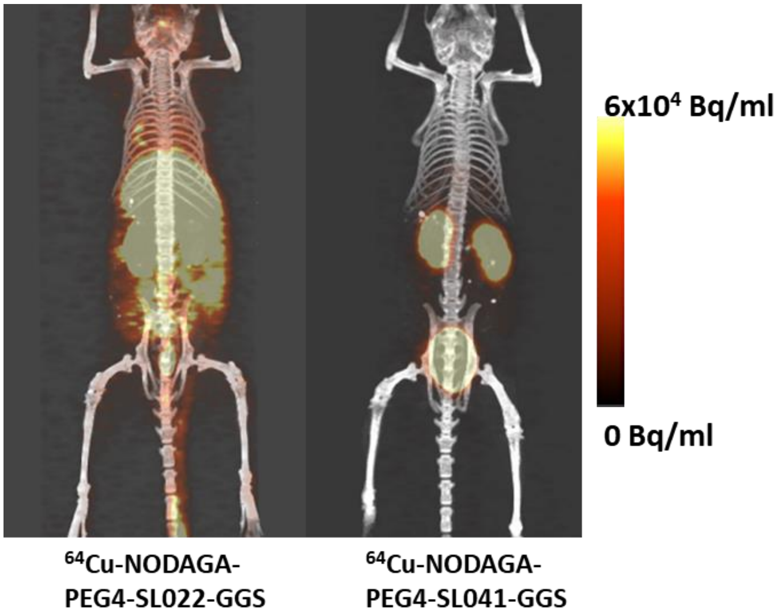

Fig. S8. Representative PET comparing target compound (^{64}Cu-NODAGA-PEG4-SL022-GGS) with the scrambled control (^{64}Cu-NODAGA-PEG4-SL041-GGS) in a disseminated MM.1S-CBR-GFP-WT xenograft model.

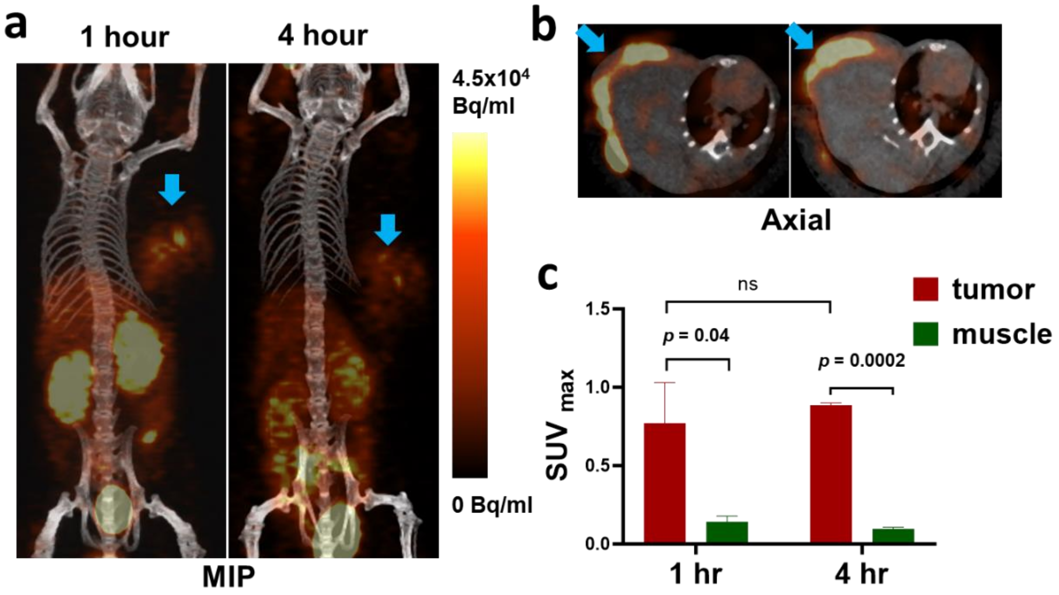

Fig. S9. Representative **(a)** MIP and **(b)** Axial PET obtained in a subcutaneous MM.1S-CBR-GFP-WT human MM xenograft mouse model. Animals are observed in the prone position. Blue arrows indicate tumor. **c)** SUV_{max} obtained from quantification of PET images. P values corrected for false discovery rate (FDR).
